# Integrated single-nucleus and spatial transcriptomics define tumour cell metaprograms and tumour microenvironment organisation in choroid plexus tumours

**DOI:** 10.1186/s13073-026-01814-8

**Published:** 2026-11-03

**Authors:** Anna Klötergens, Clara Inserte, Flavia W. de Faria, Archana Verma, Erik Schüftan, Rajanya Roy, Victor Banerjee, Thomas K. Albert, I-Na Lu, Uwe Kordes, Denise Obrecht-Sturm, Christian Thomas, Kornelius Kerl

**Affiliations:** 1 Department of Paediatric Haematology and Oncology, University Children’s Hospital Münster, Münster, Germany; 2 Institute of Medical Informatics, University of Münster, Münster, Germany; 3 Department of Paediatric Haematology and Oncology, University Medical Centre Hamburg-Eppendorf, Hamburg, Germany; 4 Institute of Neuropathology, University Hospital Münster, Münster, Germany

**Keywords:** Choroid plexus tumour, snRNA-seq, Spatial transcriptomics, Tumour microenvironment remodelling, Intertumoural heterogeneity

## Abstract

**Background:**

Choroid plexus tumours (CPTs) are rare intraventricular brain neoplasms derived from choroid plexus epithelium, predominantly affecting young children. They include benign choroid plexus papilloma (CPP), atypical CPP (aCPP), and malignant choroid plexus carcinoma (CPC) and segregate into three DNA-methylation subgroups: paediatric A (pedA), paediatric B (pedB), and adult.

**Methods:**

To resolve subtype-specific biology, we performed single-nucleus RNA sequencing on 43 CPTs and integrated 12 non-neoplastic foetal, paediatric, and adult choroid plexus references, complemented by spatial transcriptomics in a subset of 16 tumour sections and 2 normal choroid plexus samples.

**Results:**

Tumour embeddings and correlation analyses demonstrated that CPTs cluster by methylation subtype rather than WHO grade, revealing epigenetically driven relationships despite divergent histology. We identified 10 tumour metaprograms spanning epithelial differentiation, ciliogenesis, proliferation, extra cellular matrix remodelling, and stress responses. PedA and adult tumours showed relatively homogeneous program distribution dominated by differentiation or ciliation states, whereas pedB tumours exhibited marked heterogeneity with selective enrichment of hypoxia, interferon signalling, early stress response, and TGF-β signalling. Spatial mapping revealed consistent anticorrelation of these programs with ciliogenesis and differentiation programs, suggesting stress-associated dedifferentiation. Elevated stress and hypoxia scores correlated with poorer survival. PedB tumours showed enrichment of interferon- and lipid-associated myeloid populations, that carried pro-angiogenic signatures. They were spatially co-localised with hypoxic and interferon I expressing tumour areas, that were predicted to interact with myeloid cells via immunosuppressive cues, shaping a pro-angiogenic, pro-tumour environment.

**Conclusions:**

This study identifies stress- and hypoxia-associated dedifferentiation programs that are selectively enriched in pedB tumours, correlate with poor clinical outcome, and antagonise multiciliogenesis and epithelial differentiation. Their spatial co-localisation with immunosuppressive, pro-angiogenic macrophage populations links developmental arrest to microenvironmental remodelling and aggressive behaviour. These findings provide a biologically grounded framework for risk stratification and highlight hypoxia, angiogenesis and macrophage polarisation as candidate therapeutic targets in high-risk CPTs.

**Supplementary Information:**

The online version contains supplementary material available at https://doi.org/10.1186/s13073-026-01814-8.

## Background

Choroid plexus tumours (CPTs) are rare intraventricular epithelial neoplasms that originate from the choroid plexus (ChP), a secretory structure responsible for producing cerebrospinal fluid (CSF) and maintaining central nervous system (CNS) homeostasis [1]. Although CPTs account for only ~0.2% of all intracranial neoplasms, they represent up to 20% of brain tumours diagnosed during the first year of life [2]. According to the WHO classification, CPTs are divided into three histological subtypes defined by their mitotic activity and architectural features [3–5]: Choroid plexus papilloma (CPP, WHO grade 1), atypical choroid plexus papilloma (aCPP, grade 2), and choroid plexus carcinoma (CPC, WHO grade 3). While gross-total resection is often curative for CPP and many cases of aCPP, CPC remains associated with high relapse rates and poor survival despite multimodal therapy, underscoring the need for biologically informed risk stratification and novel therapeutic approaches [3, 4].

DNA methylation profiling has refined CPT classification into three major molecular subgroups: “paediatric A” (pedA), “paediatric B” (pedB), and “adult”, which correlate with patient age, anatomical location, and prognosis [5]. All CPCs and a subset of papillomas fall into the pedB subgroup, also referred to as the “paediatric high-risk” cluster, whereas CPTs classified as pedA or adult are generally less aggressive [6, 7]. However, despite the prognostic relevance of this classification, the biological mechanisms underlying aggressive behaviour of pedB tumours remain poorly understood. Except for *TP53* mutations in a proportion of paediatric cases and *TERT* promoter mutations in some adult cases, recurrent genetic drivers are rare, suggesting that epigenetic and developmental programs play central roles in CPT biology [8, 9]

The ChP epithelium itself is a developmentally patterned, multiciliated tissue whose specialised functions depend on tightly regulated transitions between proliferative progenitors, differentiating secretory epithelium, and mature ciliated cells that facilitate CSF flow [10–12]. Ciliogenesis is intimately coupled to epithelial differentiation and cell-cycle exit, and disruption of the multiciliogenesis program can provoke dedifferentiation and oncogenic growth in multiple epithelial cancers [13, 14]. In experimental CPT models, sustained oncogenic signalling such as *NOTCH1* or *MYC* represses key multiciliogenesis regulators (e.g., *GEMC1*/*MCIDAS*), induces loss of multiciliated morphology, and promotes aggressive behaviour, indicating that cilia-linked differentiation may operate as a lineage-stabilising constraint in this tumour family [15–17]. These insights raise the possibility that CPT subgroups represent distinct developmental or differentiation states, yet the malignant cell states corresponding to these lineages have not been resolved at single-cell resolution across subtypes.

The ChP is increasingly recognised as a neuroimmune interface at the blood–CSF barrier, housing resident macrophages (epiplexus/Kolmer cells), dendritic cells, and lymphocytes that mediate immune surveillance and regulate barrier homeostasis [12, 18]. In CPTs, emerging single-cell data indicate subtype-linked immune engagement and stromal remodelling within the highly vascular ChP niche [19]. Moreover, studies across multiple CNS tumour entities have demonstrated that hypoxic and inflammatory tumour cell states can actively remodel the microenvironment by promoting macrophage polarisation and angiogenesis [20, 21]. Whether comparable interactions occur in biologically aggressive CPT subgroups remains unknown.

Recent single-cell studies have begun to characterise the cellular composition of CPTs, providing important insights into tumour heterogeneity and biology. However, it remains unclear whether recurrent transcriptional cell states are conserved across molecular subgroups, how these states are spatially organised within the tumour, and whether they contribute to subtype-specific microenvironmental remodelling and clinical behaviour. Here, we integrated single-nucleus RNA sequencing (snRNA-seq) of 43 CPTs with spatial transcriptomics and independent bulk RNA sequencing (RNA-seq) validation to define conserved tumour cell states across the CPT spectrum. We hypothesised that the aggressive pedB subgroup is characterised by distinct developmental and stress-associated transcriptional programmes that shape tumour-microenvironmental interactions and contribute to its adverse clinical behaviour.

## Methods

### Human subject acquisition

In total, 45 formalin-fixed paraffin-embedded (FFPE) tumour blocks were collected from patients with the consent of patients or their legal representatives (Additional file 1: Table S1). Tissue samples were retrieved from the archives of the Institute of Neuropathology, University Hospital Münster, Germany, including cases submitted for central neuropathological review within the international CPT-SIOP trials. The FFPE blocks were collected between 2004 and 2024. Criteria for initial collection of specimens were the histological diagnosis as CPT WHO grade 1, 2 or 3 and a clear assignment to one of the three methylation classes adult, pedA or pedB. Non-neoplastic, FFPE embedded ChP tissue of 12 autopsy cases was collected from the archive of the Institute of Neuropathology, University Hospital Münster.

### Study cohorts

**Fig. 1:**
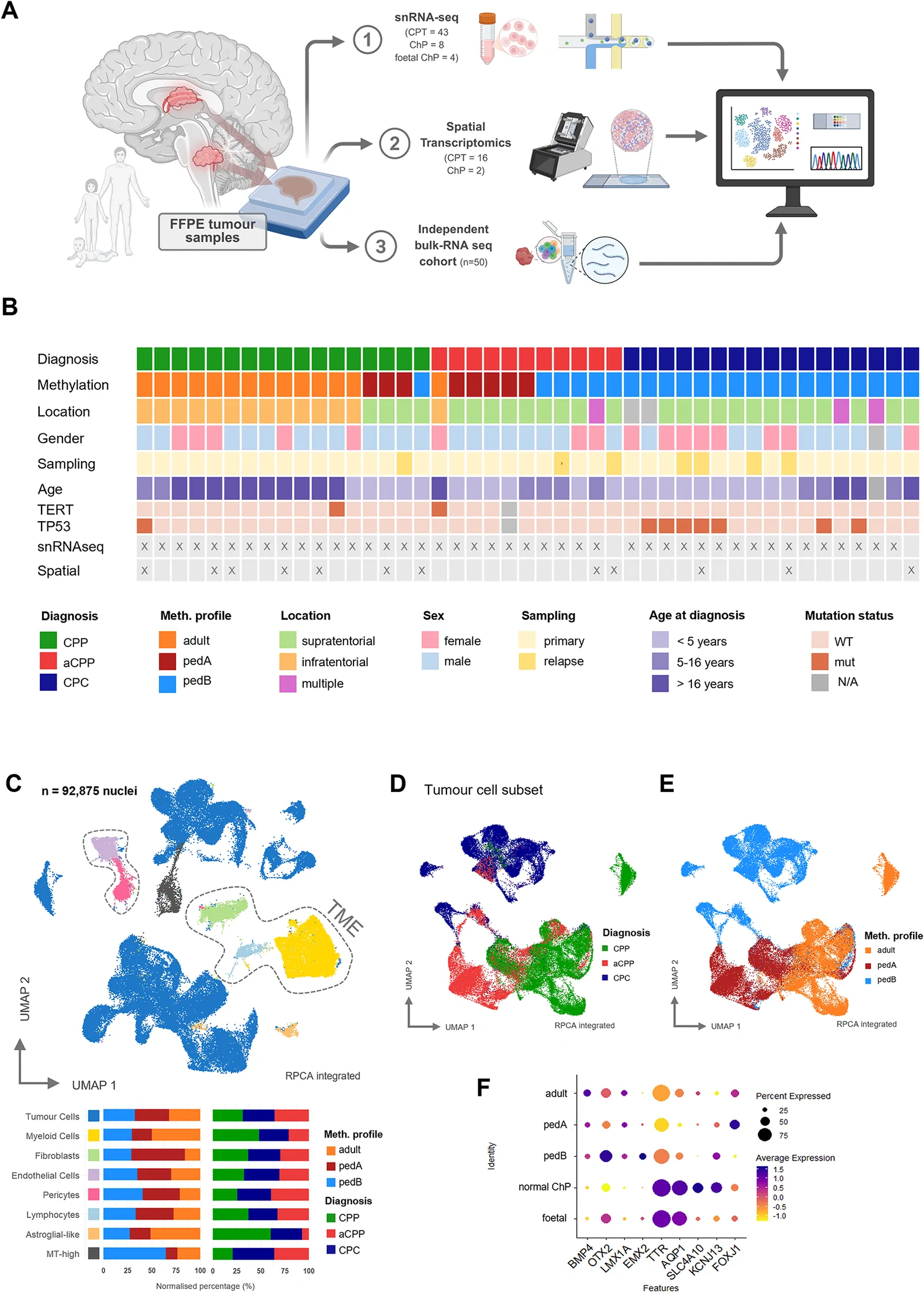
Single-nucleus transcriptomic atlas of choroid plexus tumours across histological and molecular subgroups. **A.** Study design illustrating the experimental workflow. Formalin-fixed paraffin-embedded (FFPE) tumour specimens were analysed by single-nucleus RNA sequencing (snRNA-seq) and spatial transcriptomics. An independent bulk RNA-sequencing (RNA-seq) cohort was used for validation of transcriptional programmes; *created in BioRender. Hotfilder, M. (2026)* https://BioRender.com/8j5j4u9. **B.** Overview of the study cohort showing diagnosis, DNA methylation subgroup, tumour location, patient sex, sampling status, age at diagnosis, *TP53* and *TERT* mutation status, and availability of snRNA-seq and spatial transcriptomic data. **C.** UMAP representation of 92,875 nuclei following RPCA-based integration with batch correction. Eight major cell populations were identified by functional annotation of the unsupervised clustering using canonical marker genes. Bar plots indicate the proportional distribution of histological diagnoses and DNA methylation subclasses across major cell populations. **D**, **E.** UMAP of the epithelial tumour-cell subset coloured by histological diagnosis (**D**) or DNA methylation subgroup (**E**), demonstrating stronger transcriptional segregation by molecular subgroup than by histological diagnosis. **F.** Expression of selected developmental transcription factors and epithelial differentiation markers across molecular subgroups and non-neoplastic foetal and adult choroid plexus reference cells. Average expression is colour-coded and dot size indicates the percentage of expressing cells

The study comprised five sample sets (Fig. 1a, b; Additional file 1: Table S1). (i) Tumour cohort: 45 FFPE tumour specimens from 38 patients with a histological diagnosis of CPT (17 CPP, 11 aCPP and 17 CPC) were collected at 17 centres in Germany, Belgium, Canada and Italy (centres listed in Additional file 1). All specimens were assigned to a DNA methylation subgroup (23 pedB, 8 pedA and 14 adult; see Sample validation); seven patients with divergent or uncertain classification had been excluded. Targeted next-generation sequencing was performed using a custom panel targeting *TP53* and the *TERT* promoter, as previously described [22]. The specimens included primary tumours as well as second-look or relapse operations; patient age at diagnosis ranged from 0.17 to 72 years. (ii) Non-neoplastic reference samples: FFPE ChP tissue from 12 autopsy cases (four foetal (gestational weeks 22 to 34), three paediatric and five adult) was obtained from the archive of the institute of Neuropathology, University Hospital Münster. (iii) snRNA-seq cohort: 43 of the 45 tumour specimens (36 patients; CPP *n* = 17, aCPP *n* = 10, CPC *n* = 16; pedB *n* = 21, pedA *n* = 8, adult *n* = 14), including 6 second-look or relapse operations following treatment and one case with two distinct paraffin blocks from one resection, together with the 12 non-neoplastic reference samples. (iv) Spatial transcriptomics cohort: 16 tumour sections (8 CPP, 3 sections from 2 aCPP and 5 sections from 4 CPC) and 2 non-neoplastic choroid plexus samples. (v) Immunohistochemistry cohort: 19 CPT samples (adult = 4, pedA = 5, pedB = 10, including seven CPCs). DNA methylation profiling was performed for all tumour specimens; snRNA-seq, spatial transcriptomics and immunohistochemistry were performed on the samples listed above (availability per specimen is shown in Fig. 1b and Additional file 1: Table S1).

### Independent bulk RNA-seq cohort

For independent validation, a previously published bulk RNA-seq cohort of 50 CPTs spanning all histological and methylation subgroups was analysed [23]. DESeq2-normalised [24] counts were provided by one of the original investigators (C.T.); clinical follow-up data were available for 34 samples (see Bulk RNA-seq validation).

### Sample validation

To confirm accurate determination of methylation classes, bulk methylation profiles (450k or EPIC arrays) were either generated de novo or obtained for all tumour specimens. Sample classification was performed with the Heidelberg Epignostix CNS Tumour Classifier v12.8 (https://epignostix.com/) [25]. Using this approach, 23 samples were confidently assigned to the pedB methylation subgroup, 8 to the pedA methylation subgroup and 14 to the adult subgroup (defined by a calibrated score > 0.8). For more details see Additional file 1.

### Sample preparation for single-nucleus RNA sequencing and spatial transcriptomics

For snRNA-seq, tumour samples (CPP: *n* = 17; aCPP: *n* = 10; CPC: *n* = 16) and reference samples (normal ChP: *n* = 8; foetal ChP: *n* = 4) were processed using the *Single Cell Gene Expression Flex Fixed RNA Profiling* workflow (10x Genomics, CG000632 Rev B). Spatial transcriptomics was performed on 16 CPT and 2 normal ChP tissue blocks using Visium CytAssist Spatial Gene Expression for FFPE v2 (10x Genomics). Libraries were sequenced on an Illumina NovaSeq 6000 (snRNA-seq) or NextSeq 2000 (spatial transcriptomics) platform, and the resulting data were used to analyse the spatial distribution of gene expression in the tumour samples. See detailed workflow in Additional file 1.

### Data preparation

Analysis of snRNA-seq data was performed in R (version 4.4.0), using the package Seurat (version 5.3.0) [26]. Quality control, normalisation, and feature selection were performed using standard protocols to remove low-quality cells and genes. Further steps involved reciprocal principal component analysis (RPCA) Integration [27] of all samples to correct for the 8 technical sample preparation batches, Louvain clustering [28] and dimensionality reduction of the cells with uniform manifold approximation and projection (UMAP) [29]. The resulting 26 clusters were annotated based on established cell type-specific marker genes [12, 19]. Clusters representing the same cellular identity were subsequently merged, resulting in eight major cell populations (Fig. 1c; Additional file 2: Fig. S1a). For spatial transcriptomics, raw FASTQ files from Visium FFPE libraries were processed with the 10x Genomics Space Ranger pipeline (version 2.1.0) using the “count” pipeline and the GRCh38-2020-A human reference with otherwise default settings. For further information about processing and quality control, see Additional file 1.

### Identification of tumour consensus programs by non-negative matrix factorisation (NMF)

To uncover shared transcriptional programs, we applied NMF independently to each tumour sample. Following the strategies proposed by Gavish et al. [30] and Münter et al. [31], we derived consensus programs (or metaprograms) by identifying robust sample-programs and clustering them according to shared gene content (Additional file 1). Each metaprogram was characterised by 50 representative genes (Additional file 3: Table S3). Functional annotation was performed with clusterProfiler [32] and ToppGene [33] derived Gene Ontology (GO) terms.

### Ligand-receptor analysis

Intercellular communication between tumour cells, tumour-associated macrophage (TAM) subsets, and stromal populations was analysed using the CellChat framework (version 2.0.0) [34, 35]. We employed the “CellChatDB.human” ligand–receptor database and retained interactions using default cutoff values. To integrate ligand–receptor predictions with spatial information, we overlaid CellChat-derived sender and receiver populations onto the corresponding Visium sections via their metaprogram and module scores, and qualitatively assessed whether predicted strong interactions occurred in spatially co-localised regions.

### General analysis and visualisation tools

Functional annotation and pathway enrichment analyses were performed using multiple complementary tools, including g:Profiler [36], ToppGene, clusterProfiler, and gene set enrichment analysis [37], using default parameters unless otherwise specified. Gene sets were obtained from MSigDB [38], Gene Ontology [39] and KEGG [40], and differential expression testing was performed with MAST [41] (Additional file 1). Single-cell visualisation and figure generation were facilitated by the SCpubr [42], scCustomize [43], and ggplot2 [44] R packages. Custom scripts were used for data handling, statistical summaries, and integration of results across analytical steps. AI assistance (ChatGPT 4.0/5.0, OpenAI; Claude Sonnet 5.5, Anthropic) was used exclusively for code optimisation, manuscript text refinement and manuscript formatting and did not contribute to data analysis, statistical testing, or interpretation.

### Bulk RNA-seq validation

For independent validation, we used a published bulk RNA-seq dataset of 50 CPTs spanning all histological and methylation subgroups (CPP, aCPP, CPC; pedA, pedB, adult) [23]. DESeq2-normalised counts were provided by one of the original investigators (C.T.) and imported into R. The DESeq2-normalised counts and the sample list of this cohort are provided in Additional file 4 (Tables S4 and S5). Clinical data was available for 34 samples. Digital cytometry of myeloid and TAM states from bulk RNA-seq was performed using CIBERSORTx [45]. Survival analyses were carried out in R using the survival [46] and survminer [47] packages (version 0.5.1). For more details, see Additional file 1.

### Immunohistochemistry

Immunohistochemistry was performed to validate selected transcriptomic findings at protein level. GT335 (anti-polyglutamylated tubulin) was used to assess tumour cell ciliation, Kir7.1 (potassium channel expressed in ChP epithelial cells) to validate epithelial differentiation programme and HIF1α to identify hypoxic tumour regions. Detailed staining protocols, cohort composition and scoring procedures are provided in Additional file 1.

## Results

### Cohort and study design

We conducted snRNA-seq on 43 tumour samples from 36 patients, with 6 second-look or relapse operations following treatment and 1 case with two distinct paraffin blocks from one resection. Our cohort encompasses all three histological subtypes, including CPP (*n* = 17), aCPP (*n* = 10) and CPC (*n* = 16) (Fig. 1a, b). Extending beyond the most recent single-cell study in this field [19], we selected CPP and aCPP samples from all three methylation subclasses “pedA” (*n* = 8), “pedB” (*n* = 21) and “adult” (*n* = 14). All 16 CPCs exhibited the pedB methylation profile, consistent with the established molecular classification of this tumour entity [5]. Samples were grouped according to tumour location into supratentorial (third and lateral ventricles), infratentorial (fourth ventricle), or multifocal cases (Fig. 1b). *TP53* mutations were observed in 7/16 CPCs and 1 CPP of the “adult” molecular subtype, whereas *TERT* promoter mutations were observed in 2 adult CPP (Fig. 1b). Spatial transcriptomics was performed on a total of 16 CPT sections (7 CPPs, 3 tissue sections from 2 aCPPs and 4 sections from 3 CPCs) and 2 non-neoplastic ChP samples to provide spatial context to our findings. To enable independent validation of the single-nucleus findings, we investigated a previously published bulk RNA-seq cohort comprising 50 CPTs of all histological and methylation subgroups [23] (Fig. 1a).

### Integrated snRNA-seq reveals CPT cellular architecture and subtype-linked celltype composition differences

Having established the study cohort, we first sought to define the global cellular composition of CPTs before examining tumour-cell heterogeneity across molecular subgroups. Following RPCA-based integration of all tumour samples, unsupervised clustering identified both epithelial tumour cells and non-neoplastic stromal and immune populations (Fig. 1c, Additional file 1). We annotated the major cell populations of the ChP including epithelial derived choroid ChP cells, myeloid cells, lymphocytes, fibroblasts, pericytes and endothelial cells, using marker genes from a recently published murine ChP single-cell atlas (Additional file 2: Fig. S1a)^12,19^. A small cluster of cells carried an astroglial signature (*AQP4, GFAP*), most likely reactive astrocytes from surrounding CNS tissue. The cluster annotated as “MT-high” was characterised by highly expressed mitochondrial and respiration chain genes, possibly due to low cell quality caused by necrosis, stress or technical issues. The tumour samples displayed variable proportions of the cell populations depending on histological and methylation subtype (Fig. 1c, Additional file 2: Fig. S1b). Across all groups, >70% of sequenced cells were classified as epithelial tumour cells. Tumours of the adult subgroup contained a relatively higher proportion of myeloid cells, whereas pedB tumours showed increased numbers of tumour cells with a high expression of mitochondrial genes, potentially reflecting a higher proportion of necrotic or hypoxic-stressed tumour regions.

To investigate the intertumoural differences of histological and molecular CPT subtypes, the subsequent analyses focused on the epithelial tumour cell compartment. We subset the tumour cells from the integrated object and analysed differentially expressed genes (DEGs) of the subgroups.

### Transcriptional signatures correlate with methylation subtype

Within the UMAP of the integrated tumour cell subset, all CPC samples separated from the majority of CPP and aCPP tumour cells (Fig. 1d). Histologically low-grade CPP and aCPP of the molecular high-risk pedB subgroups predominantly grouped in proximity to CPC tumour cells (Fig. 1e). The remaining CPP/aCPP samples broadly separated according to their methylation status (Fig. 1e). Consistent with the overlap of tumour cells in the UMAP projection, there was a strong concordance of gene expression profiles from tumours of the pedB subgroup, regardless of the histological subtype. The gene signature of the top 100 DEGs in CPCs was enriched in papillomas of the pedB subgroup, whereas there was no significant overlap of this signature with papillomas of the pedA or adult subgroup. Conversely, the gene signature of the top 100 DEGs of aCPP and CPP did not show significant expression in pedB CPTs (Additional file 2: Fig. S1c). This visual observation was supported by a sample-wise correlation matrix of the 2,000 most variable genes across the whole tumour cell subset, which confirmed a high degree of overlap at the transcriptional level among tumours of the pedA, pedB and adult subgroups (Additional file 2: Fig. S1d). Together, these findings show that transcriptional profiles align more closely with methylation subgroups, consistent with the well-known interplay between DNA methylation and gene transcription.

### PedB tumours transcriptionally overlap with early foetal ChP and exhibit a dedifferentiated, pro-neurogenic state

Because DNA methylation subgroup emerged as the principal determinant of tumour-cell transcriptional identity, we next asked whether these subgroup-specific transcriptional profiles correspond to distinct developmental states of the normal choroid plexus epithelium. Tumour cells were integrated with non-neoplastic foetal, paediatric, and adult ChP epithelial reference cells. When projected together, non-tumour ChP samples localised near adult and pedA tumours in UMAP space (Additional file 2: Fig. S1e). In contrast, early foetal ChP cells from gestational weeks 22 and 28 converged with pedB tumour cells, a pattern corroborated by the correlation matrix of the highly variable genes (Additional file 2: Fig. S1f). Differential gene expression analyses comparing tumour subgroups versus normal ChP identified enrichment of extracellular matrix (ECM)-associated programmes, such as Reactome pathway “Collagen chain trimerization” [48] across all three CPT subtypes, with the strongest signal in pedB, followed by pedA and adult tumours (Additional file 2: Fig. S1g). Foetal ChP tissue also showed elevated ECM signatures relative to mature ChP. Genes associated with electron transport chain (ETC) and oxidative phosphorylation were uniformly downregulated, most prominently in pedB tumours (Additional file 2: Fig. S1h). As the highly secretory ChP epithelium contains high contents of mitochondria to sustain the ATP demands of continuous CSF production [49], reduced ETC gene expression is consistent with loss of specialised epithelial function, metabolic reprogramming, and/or a generally less differentiated epithelial phenotype. Consistent with these observations, pedB cells showed enhanced expression of early ChP differentiation markers (e.g., *OTX2, LMX1A*) and ectopic pro-neuronal transcription factors involved in early embryogenesis such as *EMX2*, while exhibiting reduced expression of mature ChP differentiation markers (e.g. *FOXJ1*) (Fig. 1f). Immunohistochemical staining for Kir7.1 (*KCNJ13*), a potassium channel expressed in differentiated choroid plexus epithelium, further supported these findings, demonstrating strong membranous enrichment in adult and pedA tumours, whereas CPC and to a lesser extent pedB aCPPs showed markedly reduced staining (Additional file 2: Fig. S1i).

Together, these data position pedB tumours at a transcriptionally more dedifferentiated state within the choroid plexus epithelial maturation. Although developmental mapping explained differences between molecular subgroups, it did not address the substantial transcriptional heterogeneity observed within individual tumours. We therefore next sought to identify recurrent tumour-cell states that are shared across patients but independent of predefined molecular subgroups.

### Non-negative matrix factorisation identifies ten recurrent metaprograms capturing tumour cell states and reveals marked intratumoural diversity across CPT subtypes

**Fig. 2:**
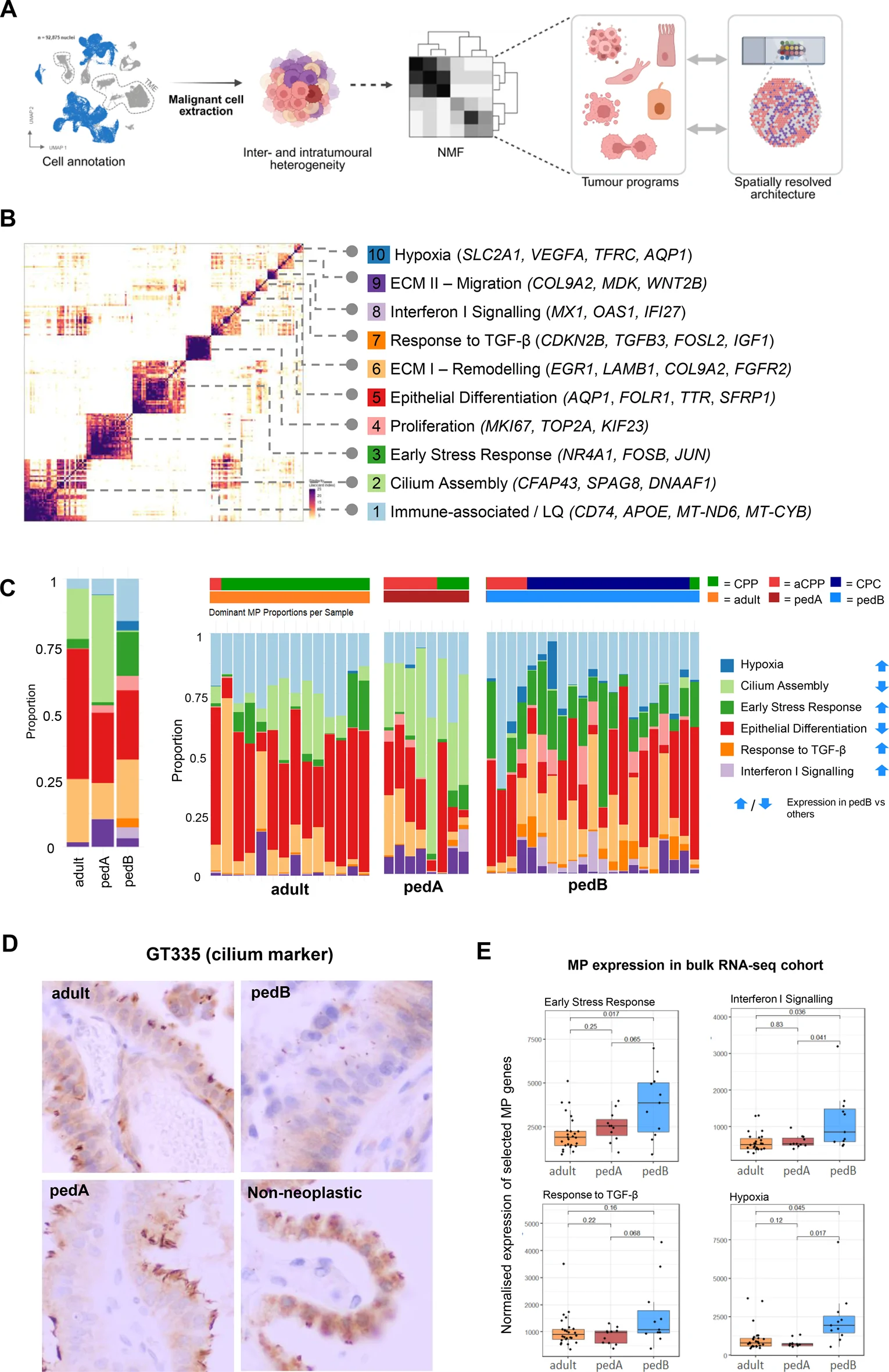
Consensus non-negative matrix factorisation (NMF) identifies recurrent tumour-cell metaprograms and reveals intratumoural heterogeneity in CPTs. **A.** Workflow of the NMF analysis. Following extraction of the epithelial tumour-cell subset, consensus NMF was performed to identify recurrent transcriptional programmes independent of cluster identity; *created in BioRender. Hotfilder, M. (2026)* https://BioRender.com/71z59tw. **B.** Consensus NMF identified ten recurrent tumour-cell metaprograms (MPs) across the epithelial tumour compartment. Functional annotations were assigned based on GO enrichment of the 50 constituent genes and are intended as descriptive annotations of the dominant biological processes represented by each metaprogram. **C.** Distribution of the dominant MP assigned to individual tumour cells, grouped by DNA methylation group (left) and individual tumour sample (right). The cilium assembly programme (MP2) was nearly absent in pedB tumours, while stress-associated MPs (MP3, MP7, MP8, MP10) were predominantly enriched in pedB tumours in variable proportions, revealing marked intratumoural heterogeneity within this subgroup. **D.** representative immunohistochemical staining for the ciliary marker GT335 across DNA methylation subgroups and non-neoplastic choroid plexus (ChP) validating subgroup-specific differences in multiciliation. Adult tumours retain moderate ciliation, pedA tumours exhibit extensive multiciliation, whereas pedB tumours show a near-complete loss of ciliary structures, validating the transcriptomic depletion of ciliogenesis-associated genes. **E.** Validation of pedB-enriched stress-associated metaprograms in the independent bulk RNA-seq cohort. Boxplots show normalized expression scores for representative genes of the early stress Response-, interferon I signalling-, Response to TGF-β- and hypoxia-metaprograms across DNA methylation subgroups. Statistical significance was assessed using wilcoxon rank-sum test

While gene expression analysis suggested pedB tumours as the most dedifferentiated group, it remained unclear whether this phenotype reflected a relatively uniform tumour-cell state or concealed additional functional heterogeneity. To identify recurrent transcriptional programmes independent of cluster identity, we applied consensus NMF to the epithelial tumour-cell compartment (Fig. 2a). Consensus NMF identified ten recurrent metaprograms (MPs) that were reproducibly detected across independent tumours (Fig. 2b). Functional annotations were assigned based on pathway enrichment of 50 constituent genes and are intended as descriptive annotations (Additional file 1).

Scoring individual tumour cells for metaprogram activity revealed marked differences in intratumoural organisation between molecular groups (Fig. 2c) Consistent with their less differentiated phenotype, the most prominent subgroup-specific difference was the near-complete absence of the ciliogenesis metaprogram in pedB tumours. Reduced expression extended beyond CPCs and was also observed in pedB papillomas, indicating that impaired multiciliation represents a molecular characteristic of the pedB subgroup rather than malignant histology alone (Additional file 2: Fig. S2a) We further assessed the expression of different functional cilia genes and observed diminished expression of all cilium- and multiciliation genes on both regulator and effector level (Additional file 2: Fig. S2b). In contrast, pedA and adult tumours retained strong expression of cilia-related genes, in many cases exceeding that observed in non-neoplastic choroid plexus tissue. These transcriptomic findings were further supported by spatial transcriptomics, which confirmed the regional distribution of cilia-associated gene expression, and by immunohistochemical staining for the ciliary marker GT335, demonstrating preserved ciliary structures in pedA and adult tumours but markedly reduced multiciliation in pedB tumours (Fig. 2d; Additional file 2: Fig. S2c). Notably, pedA tumours frequently exhibited unusually long, densely packed multicilia with a disorganised orientation, closely resembling the ciliary morphology observed in foetal choroid plexus, whereas adult non-neoplastic choroid plexus displayed shorter and more regularly organised cilia. Quantitative assessment of mono- and multiciliated epithelial cells corroborated these observations and further confirmed the marked reduction of ciliation in pedB tumours (Additional file 2: Fig. S2d). Across all tumour subgroups, metaprograms associated with epithelial differentiation (MP5), ECM organisation (MP6, MP9) and, in a subset of tumours, proliferation (MP4) represented shared biological programmes (Fig. 2c, Additional file 2: Fig. S2e). In contrast, four stress-associated metaprograms annotated as Hypoxia, Early Stress Response, Response to TGF-β and Interferon I Signalling were almost exclusively observed in pedB tumours. These programmes occurred in varying combinations and proportions across individual tumours, highlighting considerable intraclass heterogeneity despite their shared DNA methylation classification (Fig. 2c). The enrichment of stress-associated metaprograms in pedB tumours was independently confirmed in the bulk RNA-seq cohort, demonstrating that these transcriptional programmes are detected across independent patient cohorts (Fig. 2e) An additional metaprogram (MP1) comprised immune-related genes, including *APOE, CD74, CD163* and *C1QA/B*, together with elevated mitochondrial transcripts. As we could not confidently distinguish a biologically meaningful inflammatory programme from technical or tissue-quality effects such as ambient RNA contamination, we did not consider MP1 in subsequent analyses.

Together, these findings indicate that pedB tumours are characterised by a combination of developmental immaturity and pronounced functional heterogeneity. While differential gene analysis identified a less differentiated transcriptional state as a shared feature of the pedB subgroup, the NMF analysis revealed that this background is accompanied by diverse stress-associated tumour-cell programmes that vary considerably between individual tumours.

The identification of recurrent stress-associated metaprograms prompted us to investigate whether these transcriptional states occupy distinct spatial niches within the tumour microenvironment (TME). We therefore integrated spatial transcriptomic analyses to resolve their anatomical organisation and cellular context.

### Dedifferentiated regions in CPTs coincide with enrichment of stress-associated signatures and marked loss of multi-ciliation

**Fig. 3:**
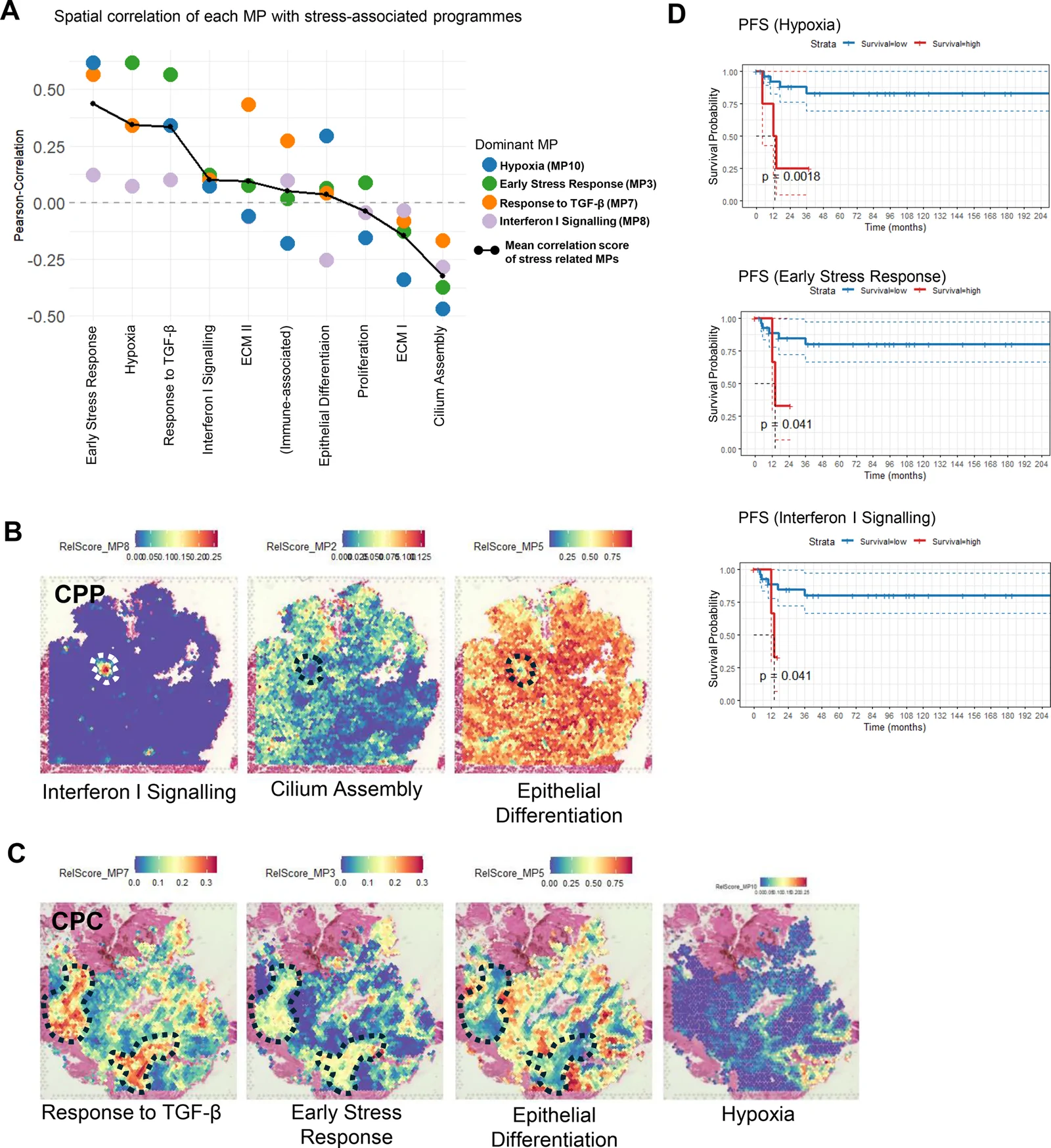
Stress-associated tumour-cell programmes occupy dedifferentiated spatial niches associated with reduced cilium gene expression and adverse clinical outcome. **A.** Spatial correlation analysis of metaprogram activity. Pearson correlation coefficients were calculated between each stress-associated MP (early stress Response, interferon I signalling, Response to TGF-β and hypoxia) and the remaining metaprograms using mean MP expression scores across spatial transcriptomics spots. Positive correlations indicate spatial co-occurrence, whereas negative correlations indicate mutually exclusive spatial organisation. **B.** representative spatial transcriptomic maps illustrating the distribution of selected MPs within a CPP. Relative MP expression score of interferon I signalling demonstrates its inverse relationship with the cilium-assembly- and Epithelial-Differentiation-program. **C.** representative spatial transcriptomic maps of a choroid plexus carcinoma (CPC). Early stress Response and Response to TGF-β exhibit marked spatial co-occurrence, whereas Epithelial-Differentiation shows a reciprocal distribution. Hypoxia is enriched in more focal tumour regions, suggesting partially independent spatial organisation of stress-associated programmes. **D.** Kaplan–Meier analysis of progression-free survival (PFS) according to hypoxia-, early stress Response- and interferon I signalling -metaprogram expression in the independent bulk RNA-seq cohort. Patients were stratified into high- and low-expression groups using maximally selected rank statistics (*maxstat*) [50]. Log-rank *p*-values are indicated

Spatial transcriptomics analyses demonstrated that the stress-associated metaprograms identified by NMF were not randomly distributed but occupied distinct niches within the tumour tissue. Analysis of spatial correlations between metaprogram signatures showed that the Early Stress Response-, Hypoxia- and Response to TGF-β-programmes were consistently co-expressed in tumour spots and high expression of Interferon I Signalling metaprogram negatively correlated with the Epithelial Differentiation-programme signature (Fig. 3a). All stress-associated metaprograms spatially anticorrelated with cilium gene expression (Fig. 3a). On an architectural level, Type-I-Interferon enriched regions in CPTs were typically organised as circumscribed “hotspots”, which spatially coincided with local loss of cilia-associated gene expression and reduced expression of the Epithelial Differentiation-associated metaprogram (Fig. 3b). While the Early Stress Response signature spatially mostly overlapped with Response to TGF-β-enriched regions that showed reduced enhancement of differentiation metaprogram, Hypoxia only partially co-occurred, suggesting that local stress-associated states can occur independently (Fig. 3c). Consistent with the spatial transcriptomic findings, immunohistochemical staining for HIF1α demonstrated focal nuclear accumulation in hypoxic regions of two CPCs, whereas no appreciable staining was detected in the more differentiated tumour subgroups (Additional file 2: Fig. S3a).

Collectively, these observations demonstrate that stress-associated metaprograms show spatial organisation and often localise to regions with reduced expression of ciliation and epithelial differentiation signatures.

### Elevated stress and hypoxia signatures associate with poor survival in CPTs

To assess the clinical relevance of these transcriptional programmes, we quantified metaprogram activity in an independent bulk RNA-seq cohort comprising 50 molecularly characterised CPTs, of which 34 had available follow-up data (mean follow-up 74 months). Cut-offs for “high” and “low” expression groups were calculated using the R package maxstat, which identified the threshold that maximises the separation between survival groups based on maximally selected rank statistics [50]. Increased Hypoxia, Early Stress Response and Type-I-Interferon-metaprogram scores were significantly associated with shorter progression-free survival (PFS) and more frequent tumour relapse (Fig. 3d). Conversely, higher Cilium programme scores showed significantly prolonged PFS (Additional file 2: Fig. S3b), consistent with the association between multiciliation and a more differentiated tumour phenotype. In addition, elevated expression of the Hypoxia programme was significantly associated with poorer overall survival (Additional file 2: Fig. S3b).

We further examined two paired pre- and post-treatment CPC samples. Whereas the patient with durable remission showed stable or reduced stress-metaprogram activity following therapy, the patient with rapid progression exhibited increased expression of Early Stress Response, Response to TGF-β, Hypoxia and Type-I-Interferon programmes after treatment (Additional file 2: Fig. S3c). Although limited to two cases, these observations illustrate that stress-associated transcriptional programmes may dynamically change during disease progression and treatment

Together, these findings identify stress-associated metaprograms as spatially organised transcriptional states that are associated with reduced epithelial differentiation and adverse clinical outcome. In previous studies, hypoxia, inflammatory signalling and TGF-β-activity have been shown to regulate macrophage biology across multiple tumour entities, which led us to investigate whether stress-enriched tumour states coincide with distinct remodelling of the immune microenvironment [51–53].

### Stress-associated tumour states are linked to an immunosuppressive and pro-angiogenic tumour microenvironment

**Fig. 4:**
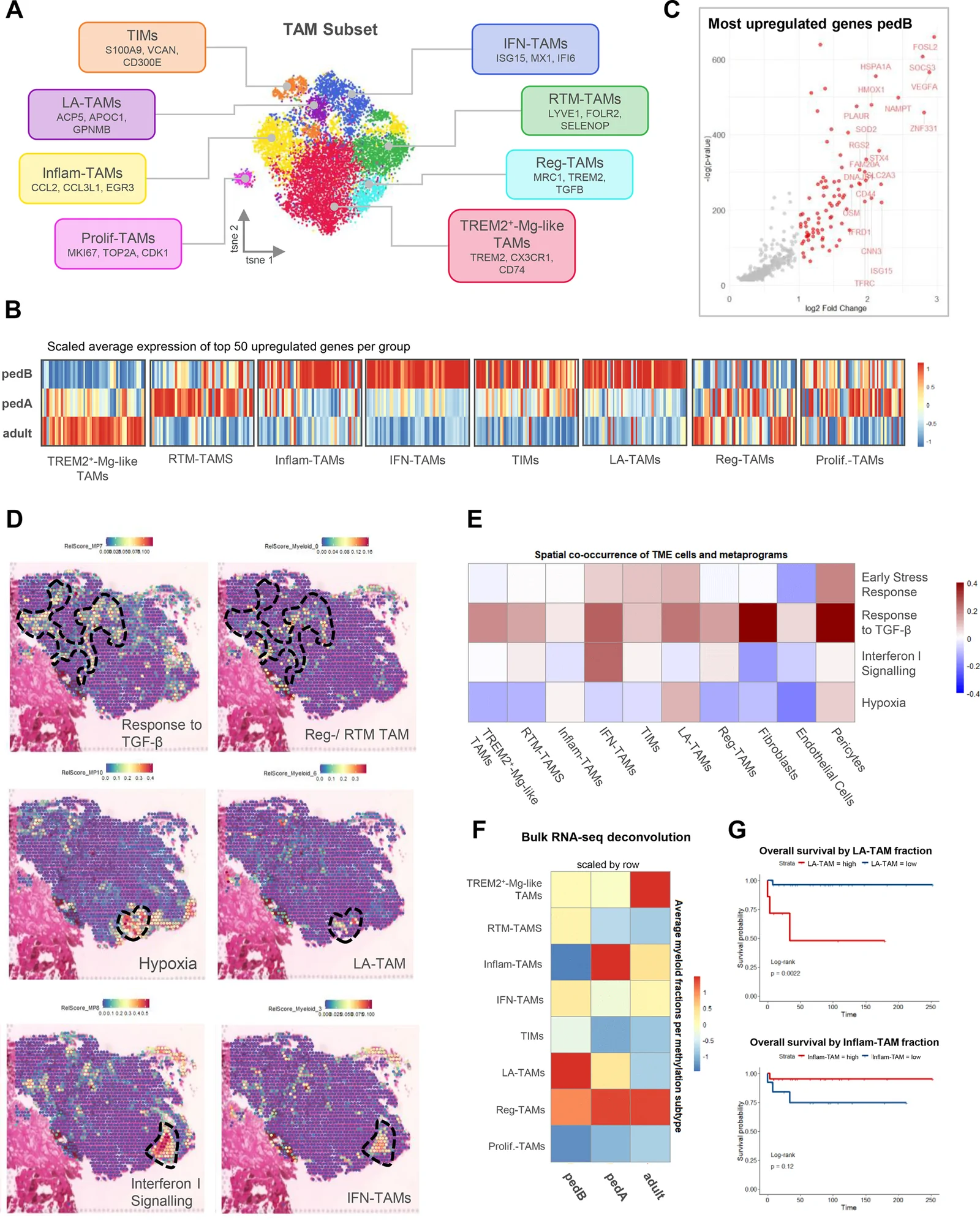
Stress-associated tumour-cell programmes spatially associate with distinct myeloid cell states and stromal remodelling. **A.** UMAP of the myeloid cell compartment following unsupervised clustering. Myeloid populations were annotated by reference mapping to the tumour-associated macrophage (TAM) atlas described by Ma et al. [54] and refined using canonical marker gene expression. **B.** Heatmap showing average expression of the top 50 marker genes defining each myeloid population. Gene expression values were scaled within each TAM population to highlight relative expression differences across DNA methylation subgroups. **C.** Volcano plot showing differential gene expression between pedB-associated myeloid cells and all remaining myeloid cells. Highlighted are the most strongly upregulated genes, several of which are associated with hypoxia, cellular stress responses and angiogenesis. **D.** Exemplar spatial transcriptomic maps of a CPC demonstrating the spatial relationship between stress-associated tumour-cell MP signature expression and distinct TAM populations. **E.** Pearson correlation analysis quantifying spatial associations between stress-associated tumour-cell MPs and TME cell populations. Positive correlations indicate preferential spatial co-occurrence of specific tumour-cell states and immune or stromal populations. **F.** Estimated abundances of TAM populations inferred from the independent bulk RNA-seq cohort using CIBERSORTx deconvolution based on single-nucleus-derived reference signatures. **G.** Kaplan–Meier analysis of overall survival according to inferred lipid-associated TAM (LA-TAM) and inflammatory TAM abundance in the bulk RNA-seq cohort. Patients were stratified into groups with high- and low-TAM fractions using maximally selected rank statistics (maxstat) [50]. Log-rank *p*-values are indicated

To determine whether tumour-cell states are associated with subtype-specific alterations of the immune microenvironment, we extracted and re-clustered all myeloid cells from the integrated dataset. The myeloid cells broadly segregated into *TREM2*^+^ macrophages with microglia-like/reactive features that spatially enriched in reactive brain tissue at the tumour border, while *LYVE1*^*+*^*/FOLR2*^+^ resident macrophages, that displayed a more anti-inflammatory, *MRC1*-associated phenotype, were localised near the vasculature or stromal niches (Additional file 2: Fig. S4a). After functional cluster annotation with canonical marker genes established by Ma et al., we identified eight distinct TAM populations [54] (Fig. 4a, Additional file 2: Fig. S4b). In comparison between the subtypes, adult tumours were enriched for *TREM2*^+^ microglia-like TAMs, whereas pedA tumours contained higher expression of resident tissue macrophage (RTM)-like TAMs likely resembling choroid plexus epiplexus macrophages (*LYVE1*^+^) [18] (Fig. 4b). PedB tumours displayed the most heterogeneous TAM landscape, with preferential expansion of interferon-responsive TAMs (IFN-TAMs), lipid-associated TAMs (LA-TAMs), and inflammatory TAMs (Fig. 4b, Additional file 2: Fig. S4c). Differential expression analysis further demonstrated increased expression of angiogenesis- and stress-associated genes including *VEGFA, FOSL2, HMOX1* and *HSPA1A* within pedB-associated TAMs (Fig. 4c).

Spatial integration further connected these TAM states to pedB tumour metaprograms: TGF-β-associated tumour regions, which co-localised with stromal and fibroblast/pericyte signatures, also co-occurred with *LYVE1*-high RTM and Immune Regulatory TAMs (Reg-TAMs) (Fig. 4d, e). Hypoxia-high tumour areas were positively associated with LA-TAMs, suggesting metabolic coupling between hypoxic tumour niches and lipid-associated macrophages. Interferon-high tumour cells spatially aligned with IFN-TAMs, indicating local reinforcement of interferon signalling across tumour–myeloid interfaces. Cell-type abundances, that were inferred from bulk RNA-seq with single-cell–derived reference signatures, independently predicted enrichment of LA-TAMs in pedB and a relative bias towards inflammatory TAMs in pedA [45] (Fig. 4f, Additional file 1). Although concordance with single-cell proportions was partial, potentially reflecting the modest myeloid contribution to bulk samples, the bulk cohort reinforced the clinical relevance of these myeloid states. Notably, higher predicted LA-TAM fractions in the bulk cohort were significantly associated with poorer survival, while inflammatory myeloid abundance associated with favourable outcome (Fig. 4g).

Finally, ligand-receptor analysis was performed to explore potential communication between tumour-cell states with their surrounding microenvironment. TGF-β-responsive tumour cells were predicted to interact with macrophages through TGF-β-signalling and spatially co-localised with fibroblast- and endothelial-rich regions exhibiting increased *FN1* expression. Hypoxic tumour cells showed prominent *VEGF*-mediated interactions with endothelial cells, while interferon-high tumour cells displayed predicted *colony-stimulating factor*-related interactions with myeloid populations (Additional file 2: Fig. S4d, e). Although these analyses are computational predictions, they identify candidate signalling pathways linking stress-associated tumour states to stromal remodelling, angiogenesis and macrophage activation that require future functional validation [55–58]

## Discussion

Using single-nucleus and spatial transcriptomics data from a large cohort of choroid plexus tumours and non-neoplastic choroid plexus, we generated a comprehensive cellular atlas spanning all histological entities and DNA methylation subgroups. While Hill et al. provided the first single-cell characterisation of CPTs, our study extends these observations in several important aspects [19]. By integrating a substantially larger cohort across all DNA methylation subgroups with spatial transcriptomics and independent bulk RNA-seq validation, we identify conserved tumour-cell metaprograms, resolve their spatial organisation within the TME, and relate these programmes to clinical outcome. Our analyses show that transcriptional programs align most strongly with CPT methylation subclasses, consistent with observations in other paediatric brain tumours where DNA methylation classes capture major transcriptional variation [59–61]. Within this framework, we identified subgroup-specific tumour-cell programmes that showed distinct spatial organisation and were associated with differences in TME composition and clinical outcome. Notably, pedB tumours (comprising all CPC, but also a proportion of CPP and aCPP [6]) displayed a less differentiated phenotype together with recurrent stress-associated metaprograms related to Hypoxia, Early Stress Response, Interferon I Signalling and TGF-β-signalling. This less differentiated transcriptional state is consistent with previous mouse models of CPC, in which tumour transcriptomes more closely resemble embryonic rather than mature choroid plexus epithelium [62]. The spatial association of these stress-linked programmes with reduced cilium gene expression and the prediction of distinct myeloid populations suggests that developmental state, local stress responses and TME interactions are linked in high-risk CPTs.

### Stress-associated metaprograms define heterogeneous high-risk pedB tumours

Consensus NMF identified ten recurrent metaprograms representing major transcriptional programmes across CPT tumour cells. While several programs, including Epithelial Differentiation, ECM Organisation and Proliferation, were detected across all molecular subgroups, pedB tumours showed variable enrichment of four stress-associated metaprograms related to Hypoxia, TGF-β -Signalling, Early Stress Response, and Interferon I Signalling. Importantly, these programmes were not uniformly present across pedB tumours but occurred in variable combinations and proportions, highlighting considerable biological heterogeneity within this molecular subgroup. This observation may partly explain why molecular pedB encompasses both clinically indolent papillomas and highly aggressive carcinomas. One additional program (MP1) was enriched for genes including *APOE*, *CD163* and *CD74*, which are typically associated with myeloid cells. While this signature may reflect biologically relevant epithelial-myeloid interactions, its interpretation remains uncertain. Similar immune-associated transcriptional programmes have been observed in other single-cell studies and require careful interpretation, as they may arise from genuine biological interactions but can also be influenced by technical factors including ambient RNA contamination or incompletely resolved doublets [63]. Because this could not be resolved with sufficient confidence in the present study, we did not investigate this program further. Future work integrating higher-resolution spatial approaches or functional analyses may help clarify its biological significance.

Spatial transcriptomics further demonstrated that the stress-associated programmes occupied distinct tumour niches rather than being uniformly distributed throughout the tumour tissue. Similar spatially restricted hypoxic and inflammatory programmes have been described in other paediatric brain tumours, including posterior fossa ependymoma, where they are associated with mesenchymal-like tumour states and stromal remodelling [64, 65]. Across all histological entities, stress-associated metaprograms consistently coincided with reduced ciliogenesis, suggesting a relationship between local stress responses and loss of differentiated epithelial identity. This observation is consistent with previous studies demonstrating that inflammatory and hypoxic signalling can impair epithelial differentiation and promote cellular plasticity in both brain and non-brain tumours [66, 67]. The organisation of interferon-high “hotspots” with focally reduced cilium gene expression in CPTs is particularly notable. Type I interferon programs can be induced by dsRNA sensing, viral mimicry, cytosolic DNA stress, or therapy-associated damage, and may either support anti-tumour immunity or paradoxically foster immune dysfunction depending on context [68, 69]. Although the underlying mechanisms remain unclear, similar suppression of ciliation by type I interferon signalling has been reported in other ciliated epithelia [70].

### Stress-associated tumour states are linked to distinct myeloid cell populations

A major advance of our work is the integration of tumour-cell states with myeloid cell annotation and spatial transcriptomics. We identified eight transcriptionally distinct myeloid populations that closely correspond to previously described TAM states [54]. In non-neoplastic ChP and many brain contexts, *TREM2*^+^ macrophages are typically reactive or phagocytic and can localise in peritumoural or damaged tissue, whereas *LYVE1*^+^/*FOLR2*^+^ macrophages represent resident, perivascular border-associated macrophages with anti-inflammatory bias [71, 72]. The observation of pedB-specific enrichment of LA-TAMs, together with their spatial coupling to hypoxia-high tumour regions, is consistent with studies in other tumour entities demonstrating that hypoxia promotes metabolic reprogramming of macrophages towards pro-angiogenic and immunosuppressive phenotypes [51, 53]. Moreover, pedB-associated TAMs showed increased expression of *VEGFA, HMOX1, FOSL2* and heat-shock genes, indicating activation of stress- and angiogenesis-related transcriptional programmes. Deconvolution of the independent bulk RNA-seq cohort confirmed preferential enrichment of LA-TAMs in pedB tumours, and higher predicted LA-TAM abundance was associated with poorer patient survival. Ligand–receptor analysis further identified candidate signalling pathways linking stress-associated tumour-cell states with the surrounding microenvironment. Although these analyses are based on computational inference, they identify candidate mechanisms through which stress-associated tumour states may contribute to stromal remodelling, angiogenesis and immune regulation in pedB tumours [55–58].

### PedB tumours occupy an earlier, less differentiated epithelial state

By integrating epithelial tumour cells with foetal, paediatric and adult choroid plexus references, pedB tumours were positioned at an earlier point along the ChP differentiation continuum compared with adult and pedA tumours, marked by upregulated ECM programmes, reduced expression of canonical CSF transport genes and ectopic expression of pro-neurogenic transcription factors. These findings are consistent with genetic studies and model-based evidence indicating that CPC biology is strongly shaped by disrupted developmental programmes despite relatively few recurrent coding alterations beyond *TP53* in a subset of tumours [23, 62, 73, 74]. More broadly, developmental state has emerged as a major organising principle across several paediatric brain tumours, including ependymoma and atypical teratoid/rhabdoid tumours, where subgroup-specific transcriptional identities similarly reflect distinct developmental trajectories [59, 61, 75, 76].

### Ciliogenesis distinguishes low- and high-risk tumours and links to differentiation

The most striking single-cell feature separating CPT subgroups in our cohort was the near-complete loss of the cilium assembly metaprogram in pedB tumours, including pedB papillomas. Cilia loss is a recognised hallmark of CPC and has been mechanistically linked to oncogenic *NOTCH* signalling repressing the *GEMC1*/*MCIDAS* multiciliogenesis axis [17]. Our data extend these observations by showing that reduced ciliogenesis is present across the broader pedB subgroup, beyond CPCs. Conversely, pedA tumours contained cilia-high states that partly exceeded non-neoplastic choroid plexus, which may reflect retention or reinforcement of a differentiated epithelial programme in these lower risk groups, while also being consistent with the fact that choroid plexus cilia programmes vary across development and ageing [77]. Across all tumour entities, ciliogenesis was inversely associated with stress-related metaprograms, indicating that reduced (multi)-ciliation accompanies stress-associated tumour-cell states. Furthermore, higher expression of cilia-associated genes correlated with favourable patient outcome, suggesting that preservation of ciliation may represent a marker of a more differentiated and less aggressive tumour phenotype, consistent with observations in other epithelial malignancies [78–80].

### Clinical implications and future directions

Previous studies have demonstrated transcriptional heterogeneity amongst CPCs, with gene expression-based subgroups showing distinct clinical courses and prognosis [81]. Consistent with these observations, increased expression of hypoxia-, interferon-, and early stress response-associated programmes was associated with inferior PFS in an independent bulk transcriptomic cohort. In addition, paired pre/post-treatment CPC cases suggested that persistence or induction of these stress-associated programmes may accompany disease progression. While these findings support a potential relationship between stress-programme activity and clinical outcome, they should be interpreted as evidence of prognostic association rather than independent prognostic causation. Future validation in larger, prospectively annotated cohorts will be required to determine whether stress-programme activity provides prognostic information beyond established molecular risk factors, including DNA methylation subgroup. Restoring (multi-)ciliogenesis by targeting *NOTCH* or reactivating *GEMC1*/*MCIDAS* has been shown to suppress CPC growth in models, and our data imply that such strategies might also stabilise pedB papillomas [17]. Likewise, the preferential enrichment of hypoxia-associated tumour states and lipid-associated macrophages identifies hypoxia, angiogenesis and macrophage polarisation as candidate therapeutic vulnerabilities [82]. However, whether modulation of these pathways can alter tumour progression remains to be established in appropriate preclinical models.

### Limitations

First, while this study’s cohort is large given the rarity of CPT, subgroup comparisons are constrained by sample size, and survival modelling relies on bulk cohorts with limited events. Second, metaprograms represent transcriptional co-variation rather than causal pathways; future perturbation studies are needed to confirm whether stress MPs actively drive cilia loss and dedifferentiation or simply co-report harsh niches. Third, spatial transcriptomics provides spot-level mixtures, limiting definitive assignment of ligand–receptor pairs to single cells. We mitigated this by integrating single-nucleus-derived programs and validating trends in bulk data, but higher-resolution spatial methods might refine cellular interactions. Finally, treatment-associated sampling was available for only two CPC patients; hence, conclusions about therapy-induced metaprogram dynamics are hypothesis-generating.

## Conclusions

Using integrated single-nucleus and spatial transcriptomics, we define subtype-specific cellular states and their spatial organisation across the spectrum of CPTs. Our analyses identify pedB tumours as a distinct molecular subgroup characterised by a less differentiated epithelial state, reduced (multi-)ciliation and heterogeneous stress-associated tumour-cell programmes that are linked to specific myeloid-cell populations and adverse clinical outcome. Together, these findings provide a comprehensive framework for understanding cellular heterogeneity and tumour–microenvironment interactions in CPTs and generate testable hypotheses for future mechanistic and therapeutic studies.

## Electronic supplementary material

Below is the link to the electronic supplementary material.

Supplementary Material 1: Supplementary Methods, including Table S1 (human tissue samples) and Table S2 (quantification of ciliation by immunohistochemistry)

Supplementary Material 2: Supplementary Figures S1-S8 (including immunohistochemistry and H&E slide images)

Supplementary Material 3: Table S3, robust NMF metaprogram gene lists

Supplementary Material 4: Table S4 (DESeq2-normalised counts of the independent bulk RNA-seq cohort) and Table S5 (sample sheet of the bulk RNA-seq cohort)

## Data Availability

Single-nucleus and spatial transcriptomic data generated in this study (raw counts and processed data) are available at the European Genome-phenome Archive under accession EGAS50000001617 (https://ega-archive.org/studies/EGAS50000001617) [83] as datasets EGAD50000002320 (single-nucleus RNA-seq) and EGAD50000002319 (spatial transcriptomics). Because the data derive from patients, access is controlled and managed by the Data Access Committee EGAC50000000765 (https://ega-archive.org/dacs/EGAC50000000765). Full-resolution whole-slide images of the spatial transcriptomics sections are available via OMERO (https://omero-imaging.uni-muenster.de/webclient/img_detail/761636/) [84]. The DESeq2-normalised counts and the sample list with clinical data of the bulk RNA-seq dataset used for validation are provided in Additional file 4 (Tables S4 and S5).

## Abbreviations

ChP: Choroid plexus
CNS: Central nervous system
CPT: Choroid plexus tumour
CPP: Choroid plexus papilloma
aCPP: Atypical choroid plexus papilloma
CPC: Choroid plexus carcinoma
CSF: Cerebrospinal fluid
DEG: Differentially expressed gene
ECM: Extracellular matrix
EGA: European Genome-phenome Archive
ETC: Electron transport chain
FFPE: Formalin-fixed paraffin-embedded
GO: Gene Ontology
IFN-TAM: Interferon-responsive tumour-associated macrophage
KEGG: Kyoto Encyclopedia of Genes and Genomes
LA-TAM: Lipid-associated tumour-associated macrophage
MP: Metaprogram
MT: Mitochondrial
NMF: Non-negative matrix factorisation
pedA: paediatric A
pedB: paediatric B
PFS: Progression-free survival
RNA-seq: RNA sequencing
RPCA: Reciprocal principal component analysis
RTM: Resident tissue macrophage
snRNA-seq: single-nucleus RNA sequencing
TAM: Tumour-associated macrophage
TME: Tumour microenvironment
UMAP: Uniform manifold approximation and projection
