# Supplementary material for: Integrated single-nucleus and spatial transcriptomics define tumour cell metaprograms and tumour microenvironment organisation in choroid plexus tumours
Source: Genome Med. 2026 Nov 3;18:172. doi: 10.1186/s13073-026-01814-8 (PMC13737775; doi:10.1186/s13073-026-01814-8)

Fig. S1

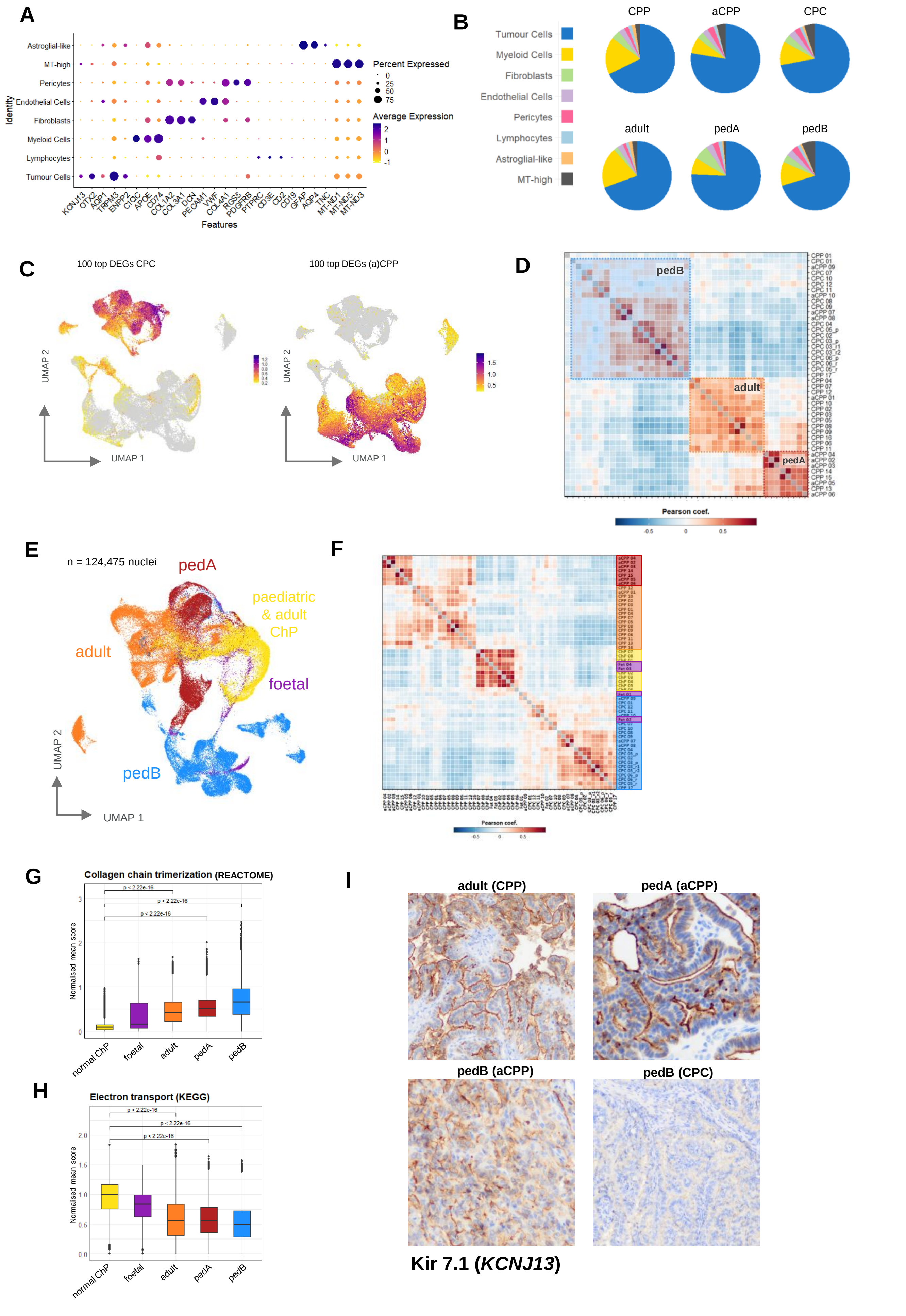

A
Astroglial-like
MT-high
Pericytes
Endothelial Cells
Fibroblasts
Myeloid Cells
Lymphocytes
Tumour Cells
Identity
Features
Percent Expressed
Average Expression
B
Tumour Cells
Myeloid Cells
Fibroblasts
Endothelial Cells
Pericytes
Lymphocytes
Astroglial-like
MT-high
CPP
aCPP
CPC
adult
pedA
pedB
C
100 top DEGs CPC
100 top DEGs (a)CPP
UMAP 1
UMAP 2
D
pedB
adult
pedA
Pearson coef.
E
n = 124,475 nuclei
pedA
paediatric & adult ChP
adult
foetal
pedB
UMAP 1
UMAP 2
F
Pearson coef.
G
Collagen chain trimerization (REACTOME)
Normalised mean score
normal ChP
foetal
adult
pedA
pedB
H
Electron transport (KEGG)
Normalised mean score
normal ChP
foetal
adult
pedA
pedB
I
adult (CPP)
pedA (aCPP)
pedB (aCPP)
pedB (CPC)
Kir 7.1 (KCNJ13)

Fig. S1 Marker-based annotation, subgroup composition and comparison of tumour cells with foetal and adult choroid plexus epithelium

A. Dot plot of canonical marker genes used to annotate the eight cell populations of the snRNA-seq atlas: tumour cells, myeloid cells, fibroblasts, endothelial cells, pericytes, lymphocytes, astroglial-like cells and cells with high mitochondrial gene expression (MT-high). Dot size indicates the percentage of nuclei expressing a gene and colour indicates average expression. **B.** Proportions of tumour and tumour microenvironment (TME) cell populations by histological diagnosis (top: CPP, aCPP, CPC) and DNA methylation subgroup (bottom: adult, pedA, pedB). **C.** UMAP feature plots showing expression of the top 100 differentially expressed genes distinguishing CPC (left) and CPP/aCPP (right). **D.** Sample-wise Pearson correlation matrix based on the 2,000 most variable genes of the malignant epithelial compartment. Unsupervised clustering groups samples primarily according to DNA methylation subgroup rather than histological identity, with pedB papillomas clustering together with CPCs while pedA CPPs and aCPPs form a separate cluster. **E.** UMAP of malignant epithelial cells integrated with non-neoplastic foetal, paediatric and adult ChP epithelial reference datasets (124,475 nuclei). PedB tumour cells show partial transcriptional alignment with early foetal ChP (gestational weeks 22 and 28), whereas pedA and adult tumours overlap predominantly with postnatal and adult ChP epithelial cells. **F.** Sample-wise Pearson correlation matrix based on the 2,000 most variable genes of malignant epithelial tumour samples together with foetal, paediatric and adult non-neoplastic ChP epithelial reference samples. Unsupervised hierarchical clustering demonstrates preferential clustering of pedB tumours with early foetal ChP reference samples (gestational weeks 22 and 28) **G.** Normalized expression of genes belonging to the Reactome “Collagen chain trimerization” pathway, identified by gene set enrichment analysis (GSEA) of genes downregulated in non-neoplastic ChP compared with tumour samples. Statistical significance was assessed using Wilcoxon rank-sum test. **H.** Normalized expression of genes belonging to the KEGG oxidative phosphorylation/electron transfer pathway, identified by GSEA of genes upregulated in non-neoplastic ChP compared with tumour samples and displayed across CPT methylation subgroups. Statistical significance was assessed using Wilcoxon rank-sum test. **I**. Representative Kir7.1 (*KCNJ13*) immunohistochemistry across DNA methylation subgroups. Strong membranous Kir7.1 expression is preserved in adult and pedA tumours, whereas pedB tumours show markedly reduced to no staining. PedB aCPPs frequently exhibited regional variation in 7.1 staining intensity.

Fig. S2

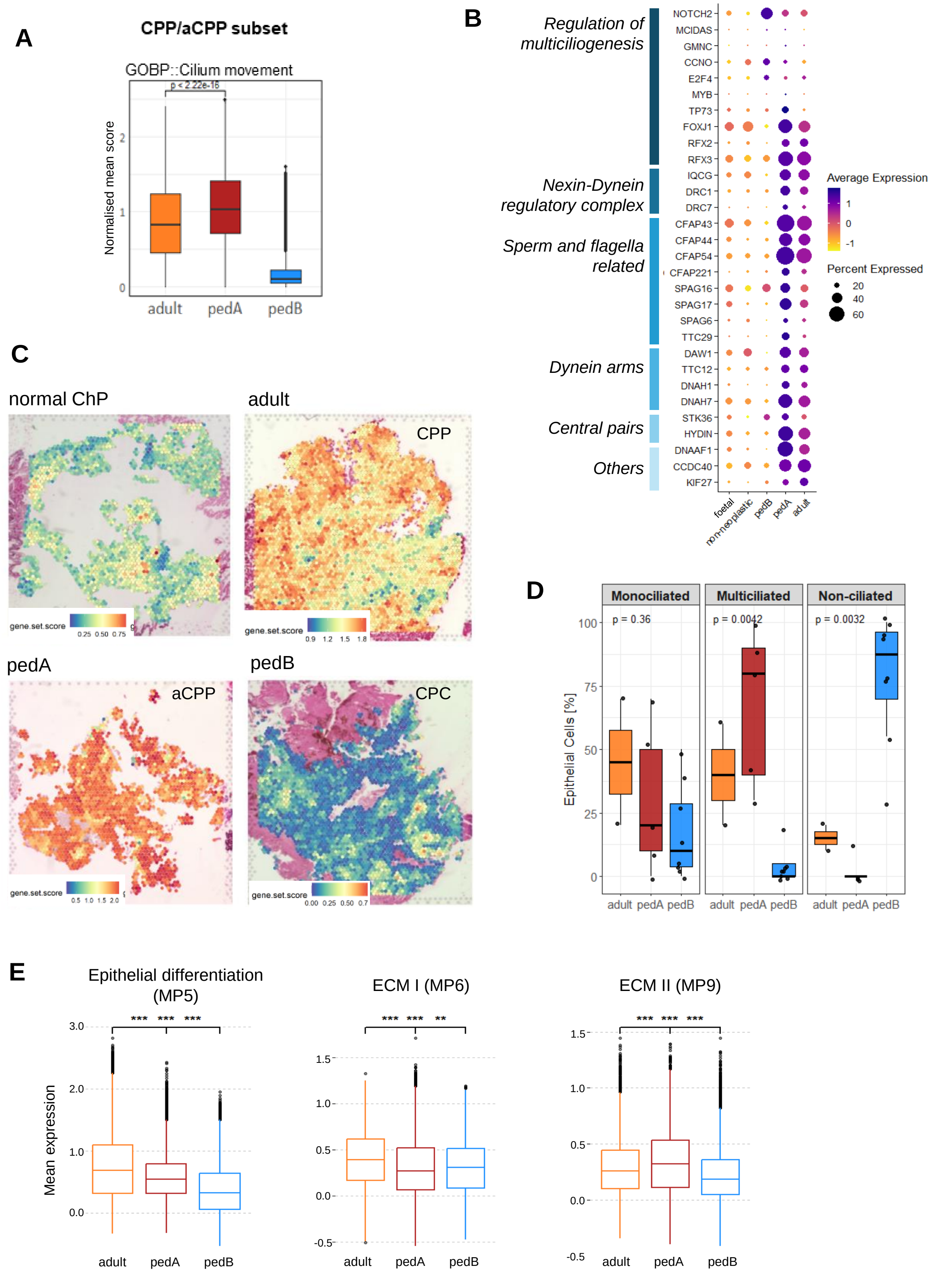
A
CPP/aCPP subset
GOBP::Cilium movement
p < 2.22e-16
Normalised mean score
adult
pedA
pedB
B
Regulation of multiciliogenesis
Nexin-Dynein regulatory complex
Sperm and flagella related
Dynein arms
Central pairs
Others
NOTCH2
MCIDAS
GMNC
CCNO
E2F4
MYB
TP73
FOXJ1
RFX2
RFX3
IQCG
DRC1
DRC7
CFAP43
CFAP44
CFAP54
CFAP221
SPAG16
SPAG17
SPAG6
TTC29
DAW1
TTC12
DNAH1
DNAH7
STK36
HYDIN
DNAAF1
CCDC40
KIF27
foetal
non-neoplastic
pedB
pedA
adult
Average Expression
Percent Expressed
C
normal ChP
adult
CPP
pedA
aCPP
pedB
CPC
gene.set.score
D
Monociliated
Multiciliated
Non-ciliated
p = 0.36
p = 0.0042
p = 0.0032
Epithelial Cells [%]
adult pedA pedB
E
Epithelial differentiation (MP5)
ECM I (MP6)
ECM II (MP9)
Mean expression
adult
pedA
pedB

Fig. S2 Ciliogenesis and epithelial differentiation programmes across DNA methylation subgroups

A. Normalised expression score of the Gene Ontology biological process (GO:BP) “cilium movement” gene set across DNA methylation subgroups. CPC samples were excluded to determine whether reduced ciliogenesis represents a molecular characteristic of the pedB subgroup independent of malignant histology. **B**. Dot plot of representative genes involved in ciliogenesis and multiciliation, grouped by function (regulation of multiciliogenesis, nexin–dynein regulatory complex, sperm and flagella related, dynein arms, central pairs, others), across foetal and non-neoplastic ChP reference cells and the pedB, pedA and adult tumour subgroups. Dot size indicates the percentage of expressing cells and colour denotes average gene expression. **C** Spatial expression scores of representative cilia-associated genes across spatial transcriptomic samples. Colour scales are shown individually for each sample. **D**. Quantification of GT335 immunohistochemical staining. Box-and-jitter plots show the percentages of monociliated, multiciliated, and non-ciliated epithelial cells in the adult, pedA, and pedB subgroup. Each dot represents one sample. P values indicate overall group differences determined by the Kruskal–Wallis test. **E**. Mean expression of the epithelial differentiation (MP5), ECM I (MP6) and ECM II (MP9) metaprogram signatures in adult, pedA and pedB tumours. Boxes represent the distribution of cell-wise expression. Asterisks indicate statistical significance.

Fig. S3

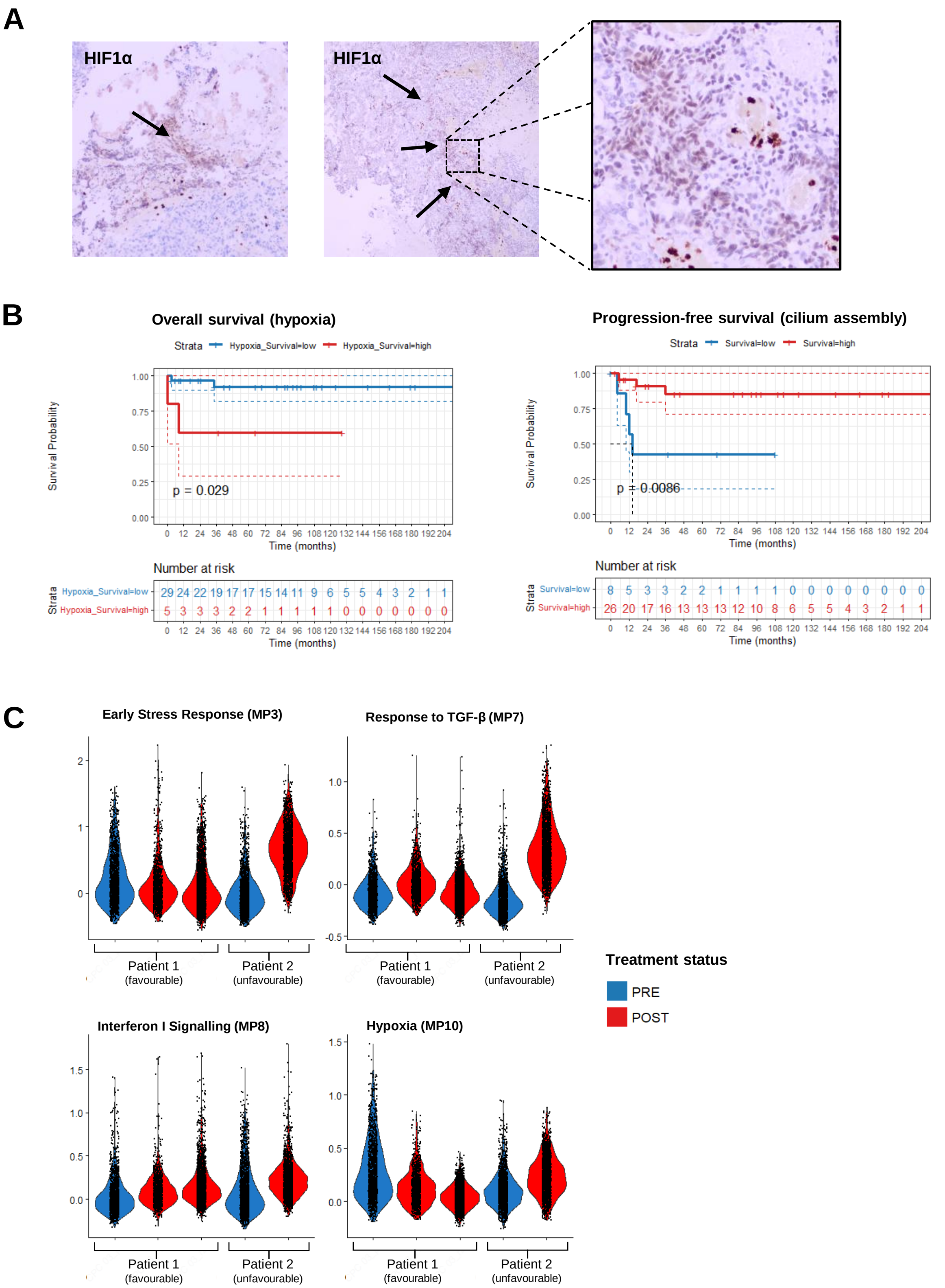
A
HIF1α
HIF1α
B
Overall survival (hypoxia)
Strata
Hypoxia_Survival=low
Hypoxia_Survival=high
Survival Probability
p = 0.029
Time (months)
Number at risk
Progression-free survival (cilium assembly)
Survival=low
Survival=high
p = 0.0086
C
Early Stress Response (MP3)
Response to TGF-β (MP7)
Interferon I Signalling (MP8)
Hypoxia (MP10)
Patient 1
(favourable)
Patient 2
(unfavourable)
Treatment status
PRE
POST

Fig. S3 HIF1α staining, survival analysis and treatment-associated changes of stress-associated metaprograms

A. Representative *HIF1α* immunohistochemical staining of two CPC samples. Focal nuclear *HIF1α* staining is detected in discrete tumour regions. The right panel shows a higher-magnification image of the indicated area. Black arrows indicate areas with *HIF1α*-positive nuclei. **B**. Kaplan–Meier analyses of overall survival (OS) according to hypoxia metaprogram (MP10) expression and progression-free survival (PFS) according to Cilium Assembly metaprogram (MP2) in the independent bulk RNA-sequencing cohort (n = 34 patients with available follow-up data). Patients were stratified into high- and low-expression groups using maximally selected rank statistics (*maxstat*)[50]. **C**. Violin plots of the activity scores of the Early Stress Response (MP3), response to TGF-β (MP7), Interferon I Signalling (MP8) and Hypoxia (MP10) metaprograms in tumour cells before (PRE) and after (POST) treatment in two paired CPC cases. Patient 1 had a favourable and patient 2 an unfavourable clinical course

Fig. S4

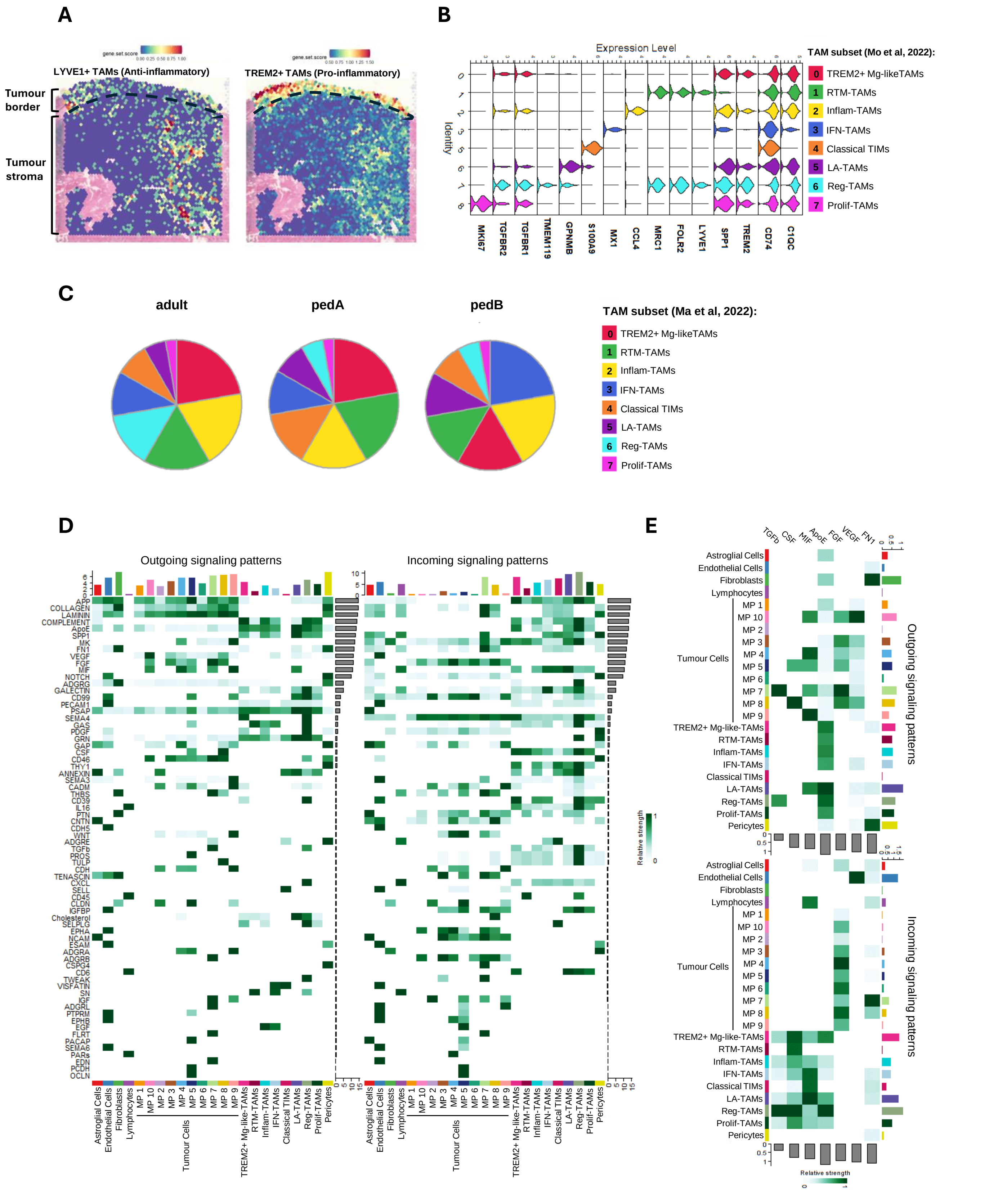
A
B
C
D
E
LYVE1+ TAMs (Anti-inflammatory)
TREM2+ TAMs (Pro-inflammatory)
Tumour border
Tumour stroma
Expression Level
Identity
TAM subset (Mo et al, 2022):
TREM2+ Mg-likeTAMs
RTM-TAMs
Inflam-TAMs
IFN-TAMs
Classical TIMs
LA-TAMs
Reg-TAMs
Prolif-TAMs
MKI67
TGFBR2
TGFBR1
TMEM119
GPNMB
S100A9
MX1
CCL4
MRC1
FOLR2
LYVE1
SPP1
TREM2
CD74
C1QC
adult
pedA
pedB
TAM subset (Ma et al, 2022):
Outgoing signaling patterns
Incoming signaling patterns
Relative strength
Astroglial Cells
Endothelial Cells
Fibroblasts
Lymphocytes
Tumour Cells
Pericytes
TGFb
CSF
MIF
ApoE
FGF
VEGF
FN1

Fig. S4 Tumour-associated macrophage subsets and CellChat-predicted interactions with stress-associated tumour cell programmes

A. Exemplar spatial transcriptomics maps (CPC 13) showing the distribution of a *TREM2*$^+$-TAM signature (pro-inflammatory ; enrichment in tumour border) and a *LYVE1*$^+$-signature (pro-inflammatory; enrichment in stromal regions within tumour). **B.** Violin plots showing expression of canonical marker genes across the eight myeloid cell populations, annotated according to the tumour-associated macrophage (TAM) subsets of Ma et al[54]: 0, TREM2$^+$ microglia-like (Mg-like) TAMs; 1, resident tissue macrophage (RTM)-like TAMs; 2, inflammatory (Inflam-) TAMs; 3, interferon-responsive (IFN-) TAMs; 4, classical tumour infiltrating monocytes (TIMs); 5, lipid-associated (LA-) TAMs; 6, immune regulatory (Reg-) TAMs; 7, proliferating (Prolif-) TAMs. **C**. Relative abundance of the identified myeloid cell populations across the adult, pedA and pedB tumours; colours as in B. **D**. Heatmaps of outgoing (left) and incoming (right) signalling patterns predicted by CellChat[34,35] for each signalling pathway (rows) and cell population (columns): tumour cells grouped by dominant metaprogram (MP1–MP10), TAM subsets and stromal populations. Colour indicates relative signalling strength. **E**. Selected CellChat-predicted signalling pathways (outgoing, top; incoming, bottom) chosen for their potential relevance to interactions between stress-associated tumour-cell programmes and the TME. Colour indicates relative signalling strength.

Fig. S5

GT335 Immunohisto-chemistry

1-2: adult
3-7: pedA
8-15: pedB

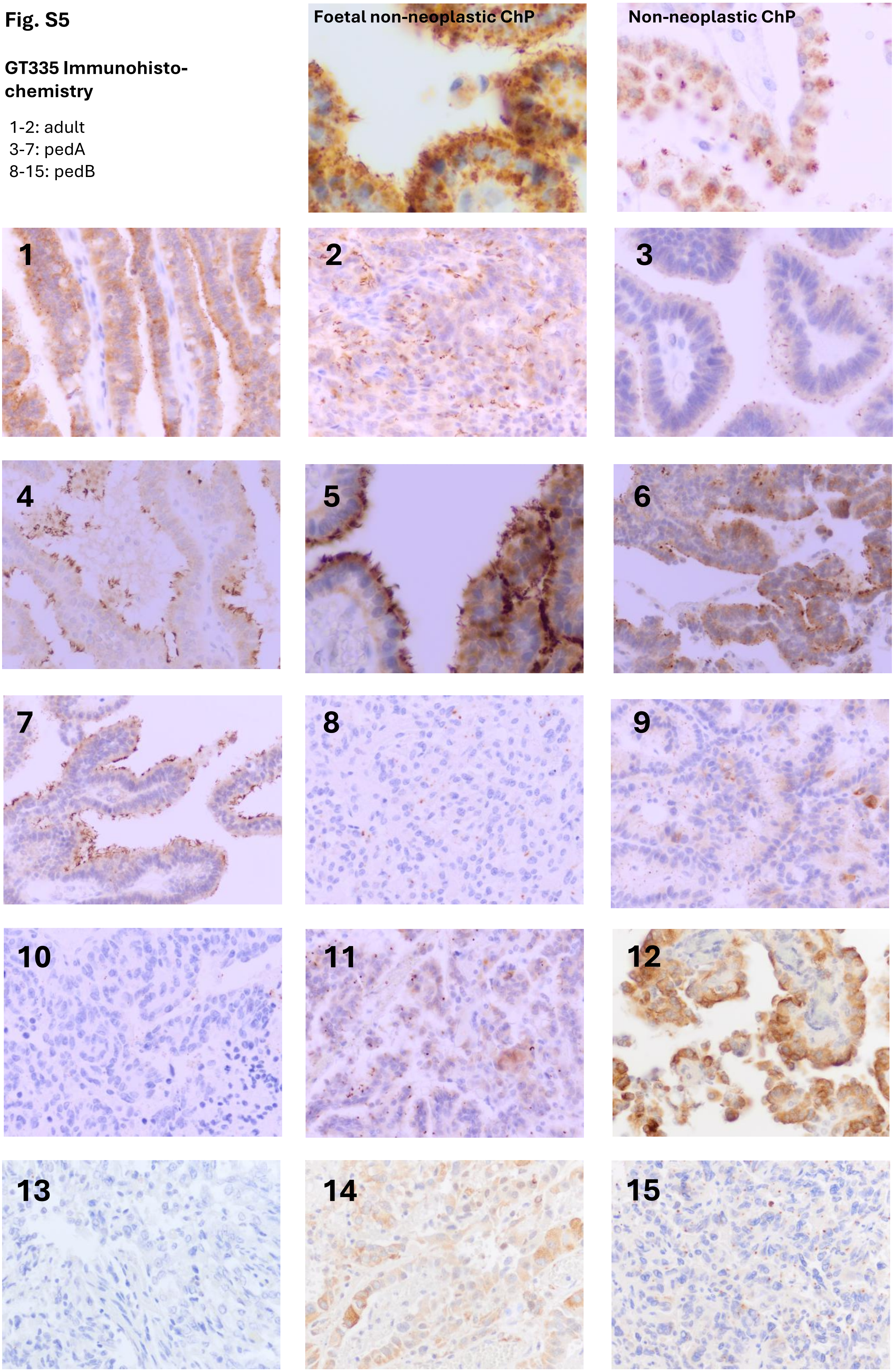

Fig. S6: Kir7.1 Immunohistochemistry

adult

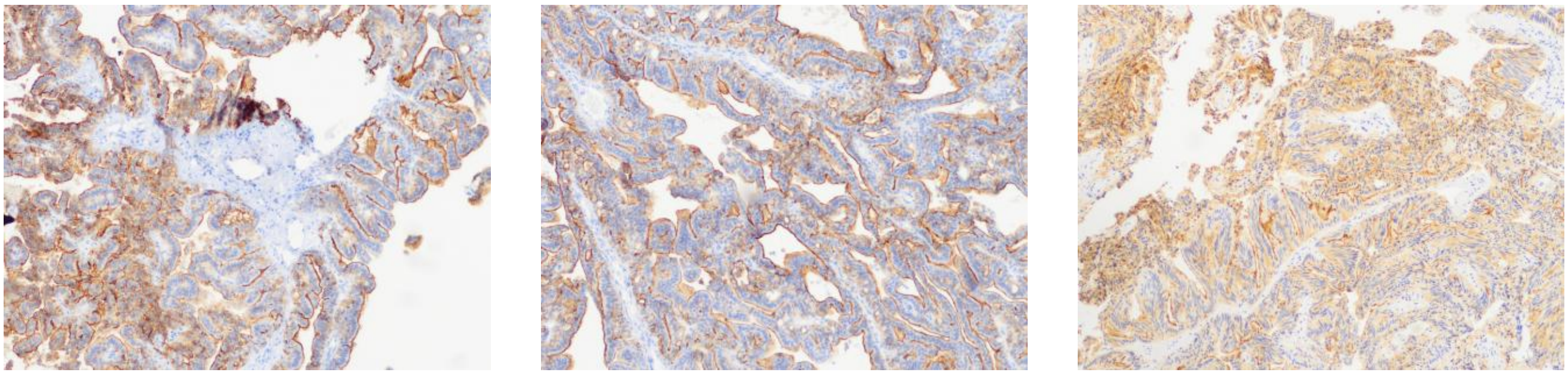

pedA

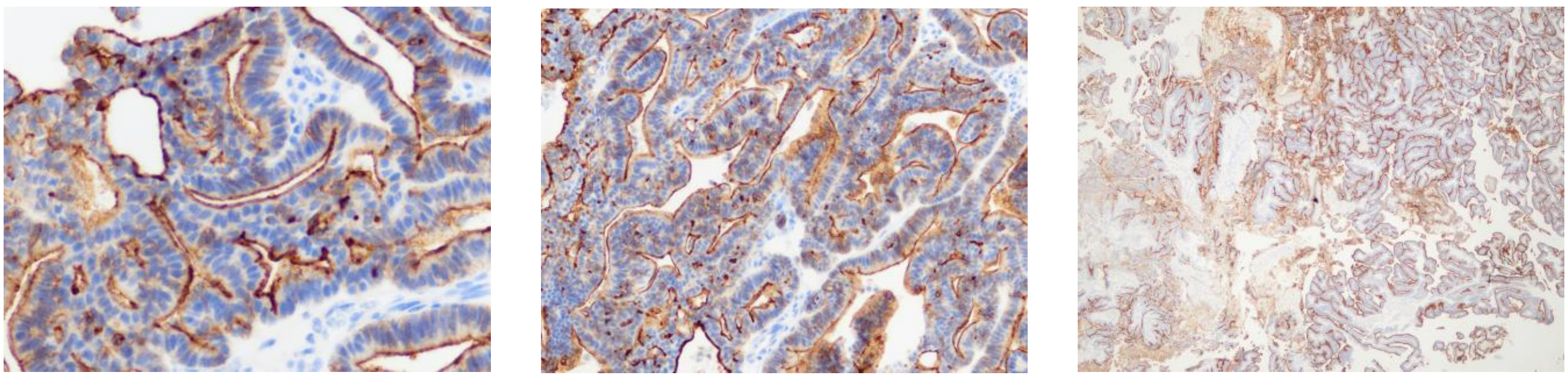

pedB

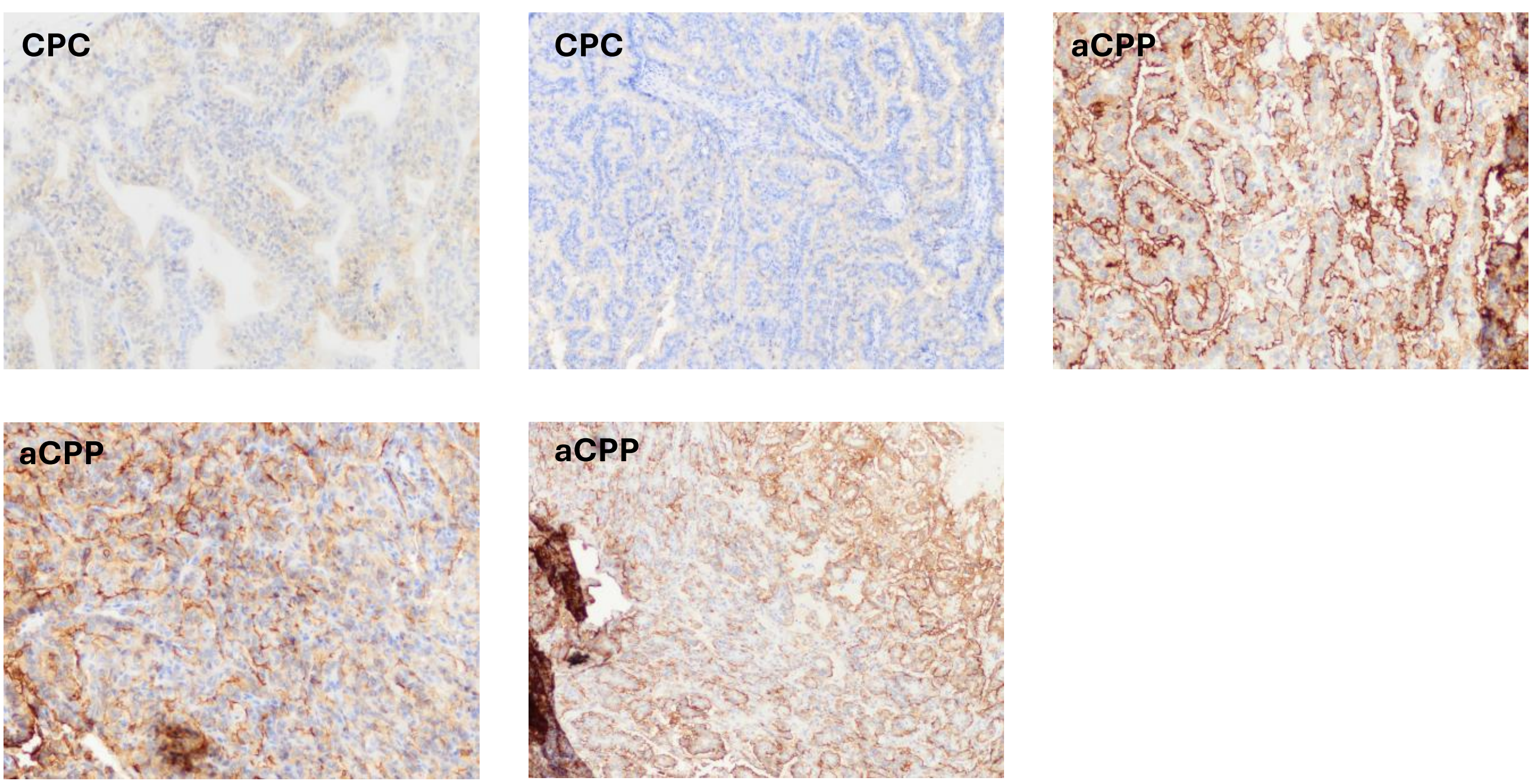

Fig. S7: HIF1α Immunohistochemistry

adult

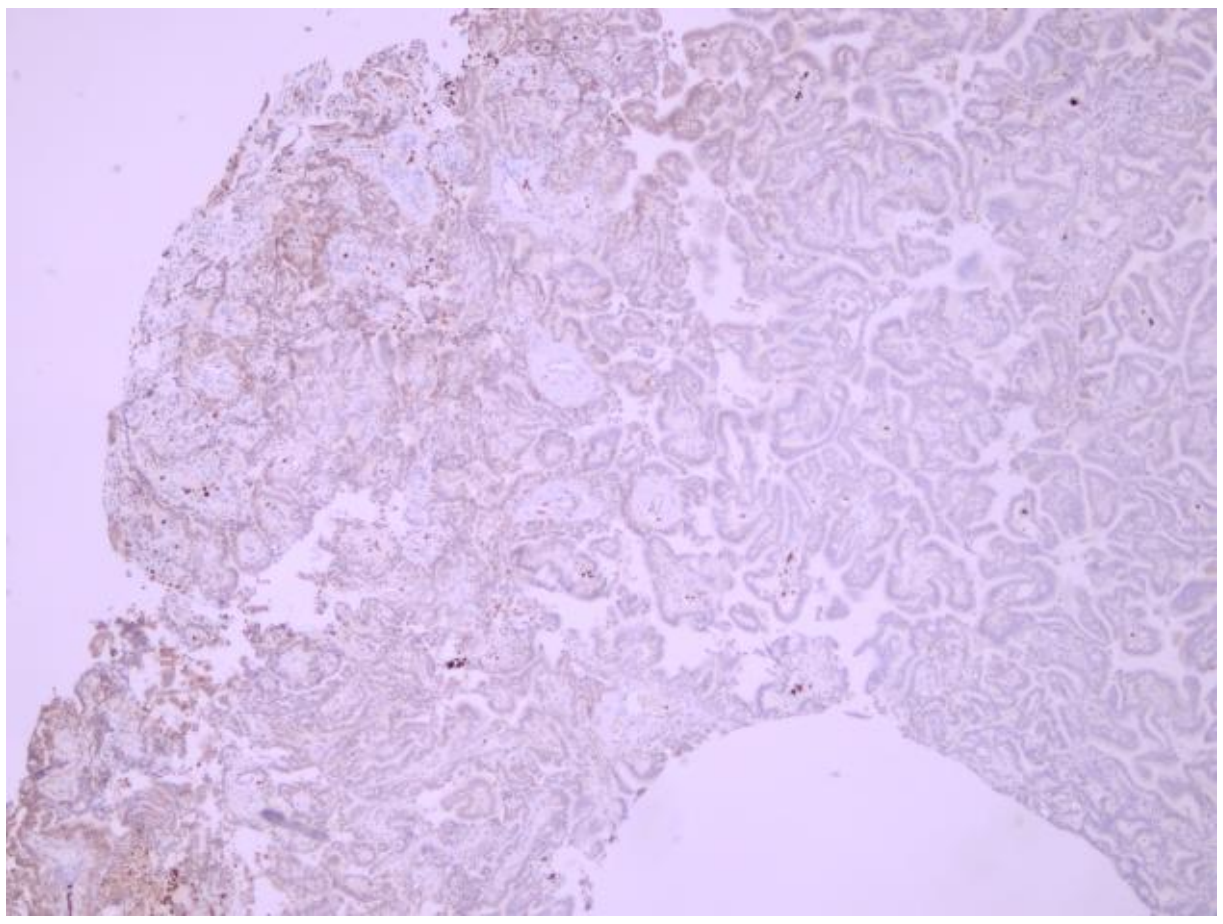

pedA

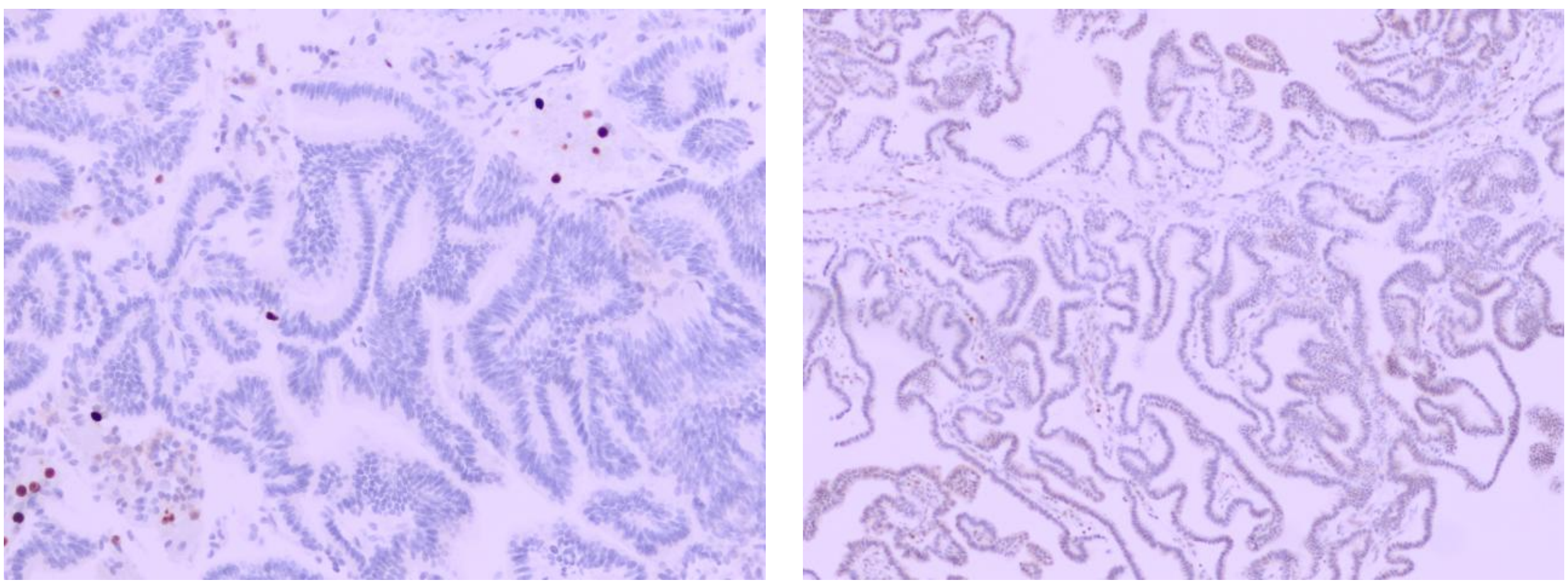

pedB

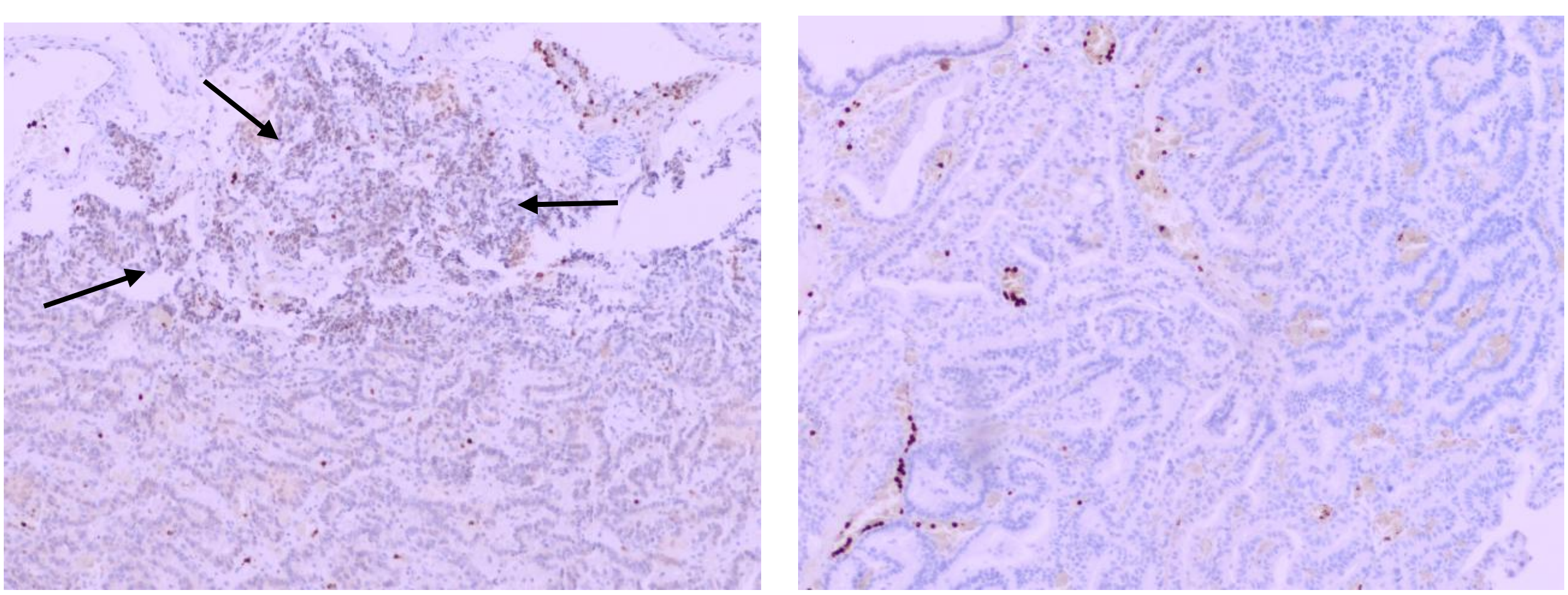

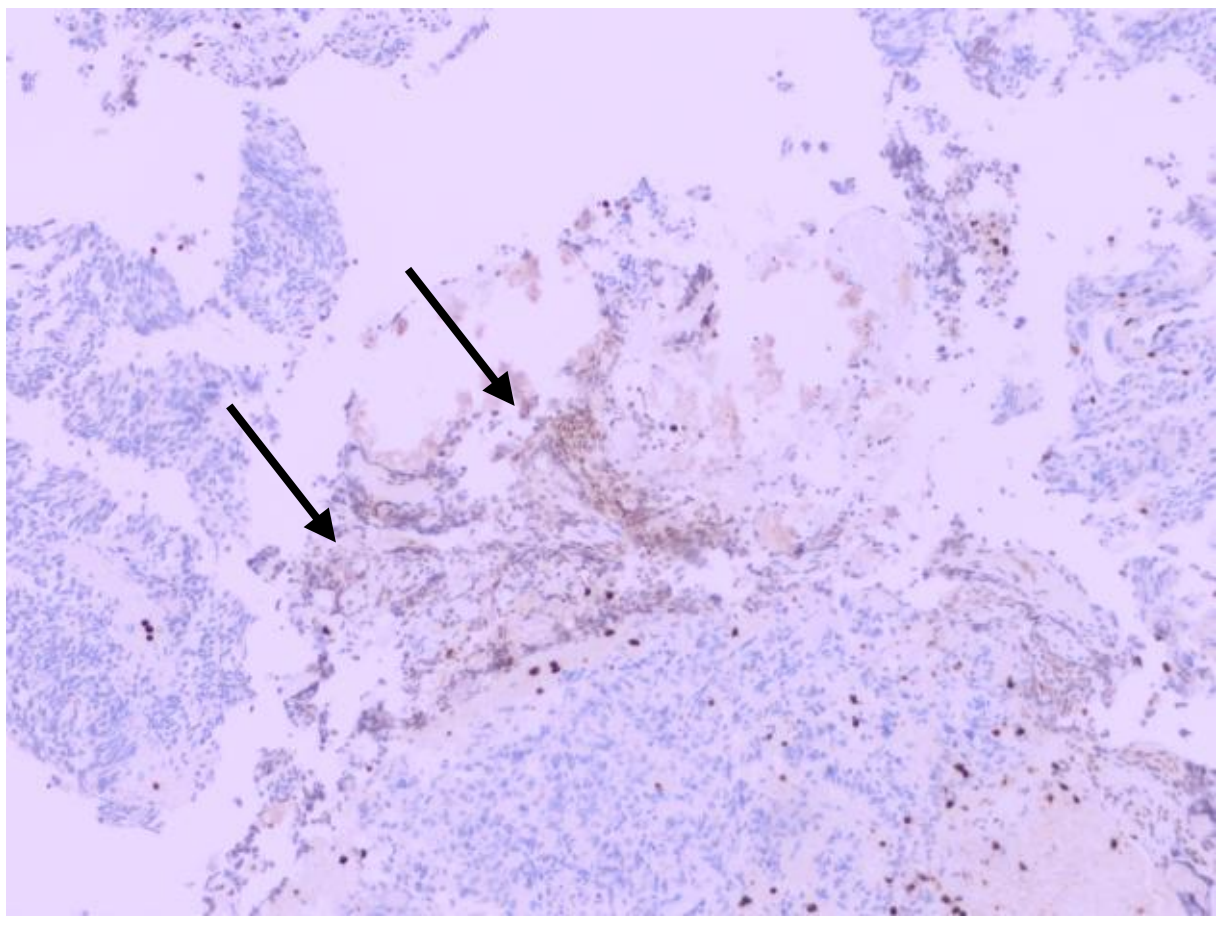
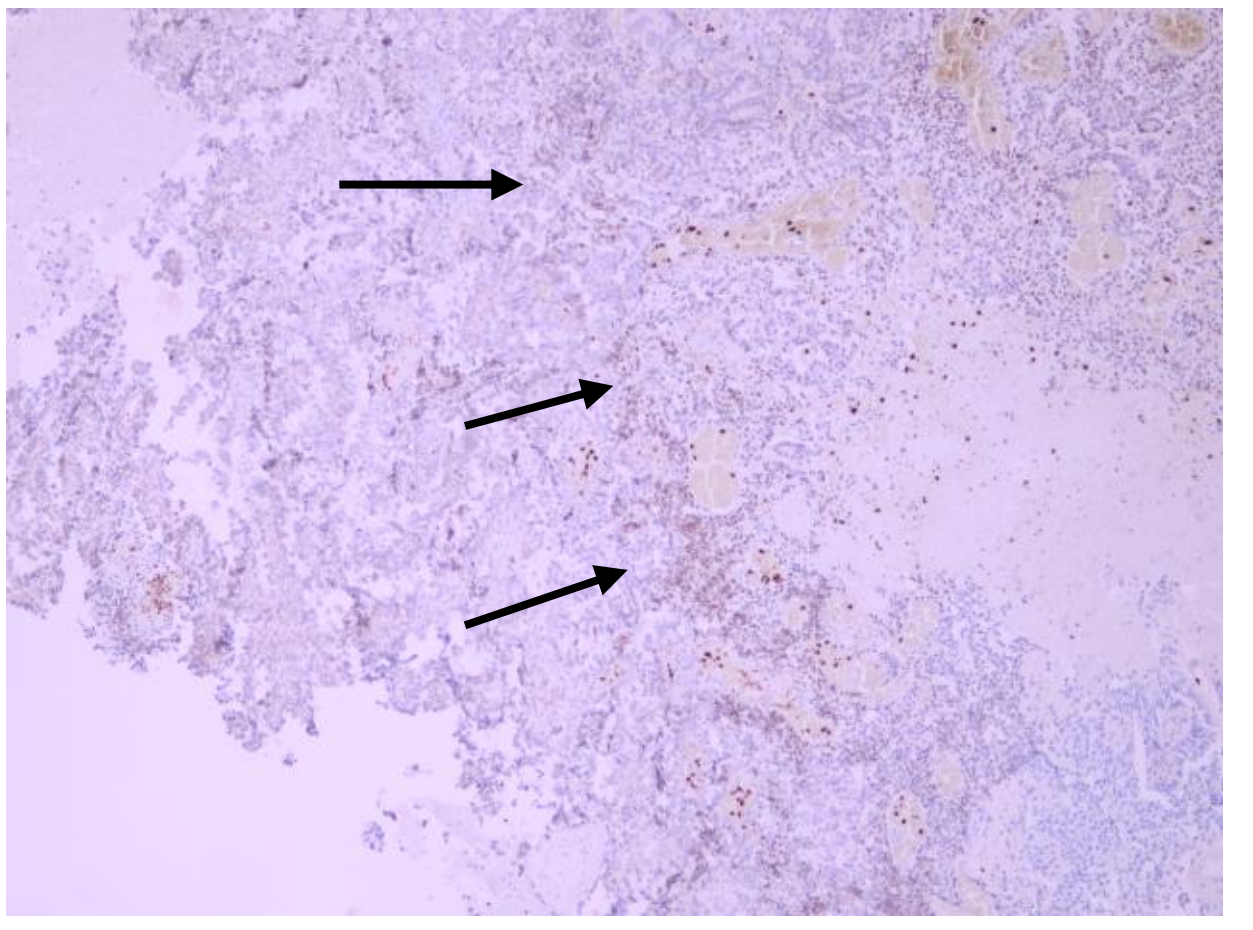
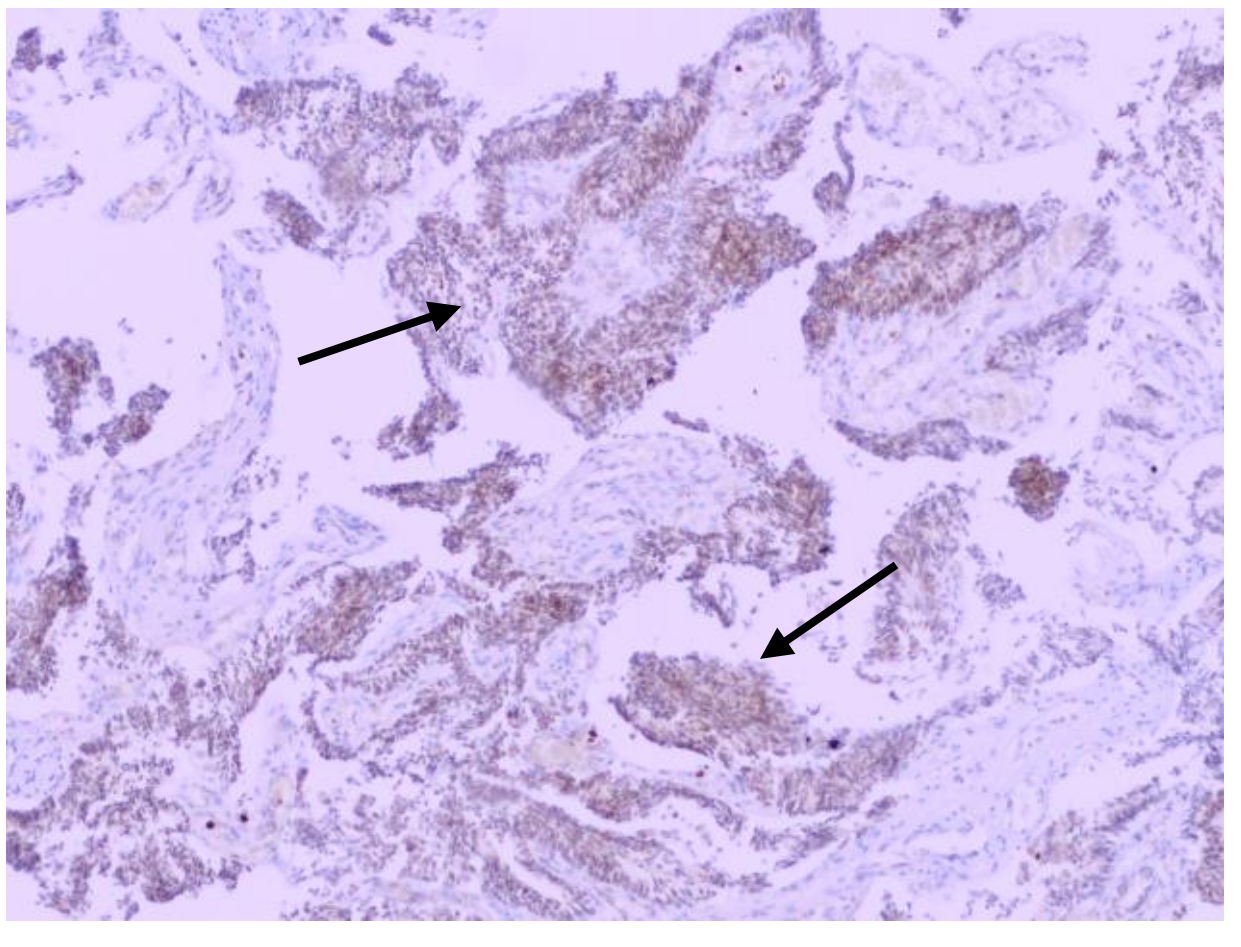

Fig. S8: HE-Images Spatial Transcriptomics

ST1 - aCPP 02
ST2 - CPP 01
ST3 - CPC 01
ST4 - CPP 05
ST5 - CPC 05_p_1
ST6 - CPC 05_p_2
ST7 - CPP 15
ST8 - CPP 17
ST9 - CPP 16
ST 10 - CPP 09
ST 11 - CPP 11
ST 12 - CPP 06
ST 13 - CPC 08
ST 14 - aCPP 11_1
ST 15 - aCPP 11_2
ST 16 - CPC 13
ST 17 - ChP 01
ST 18 - ChP 08

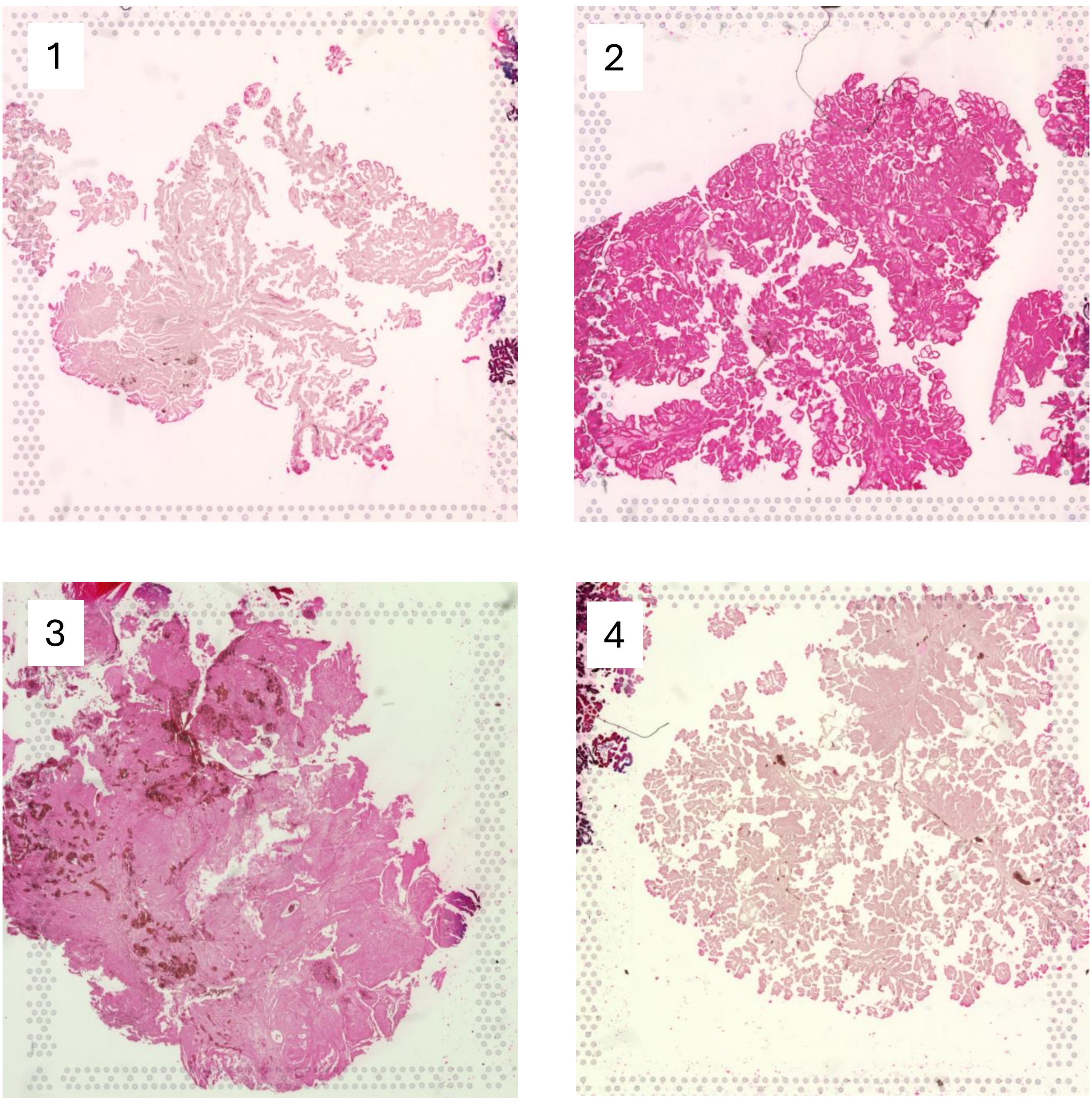

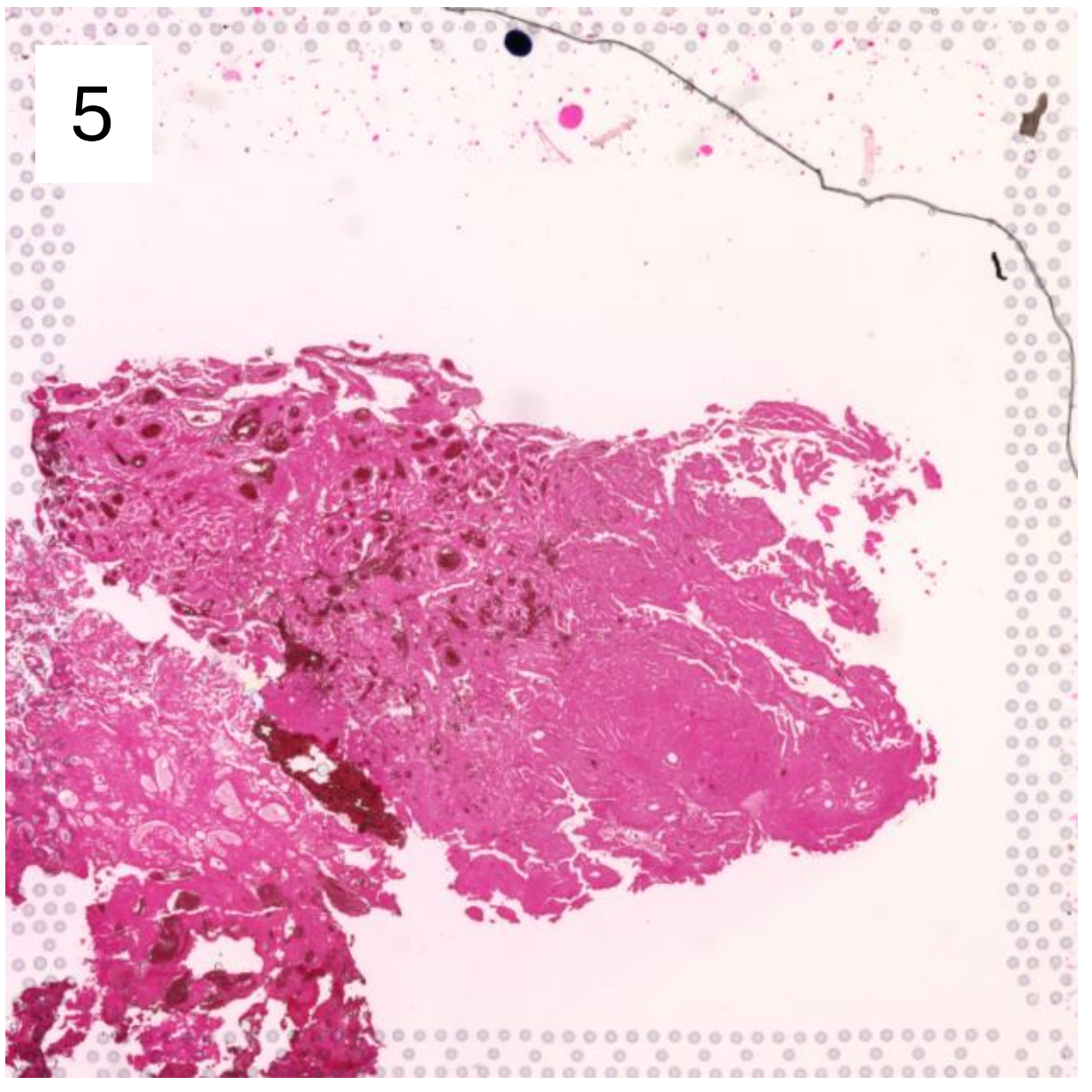
5
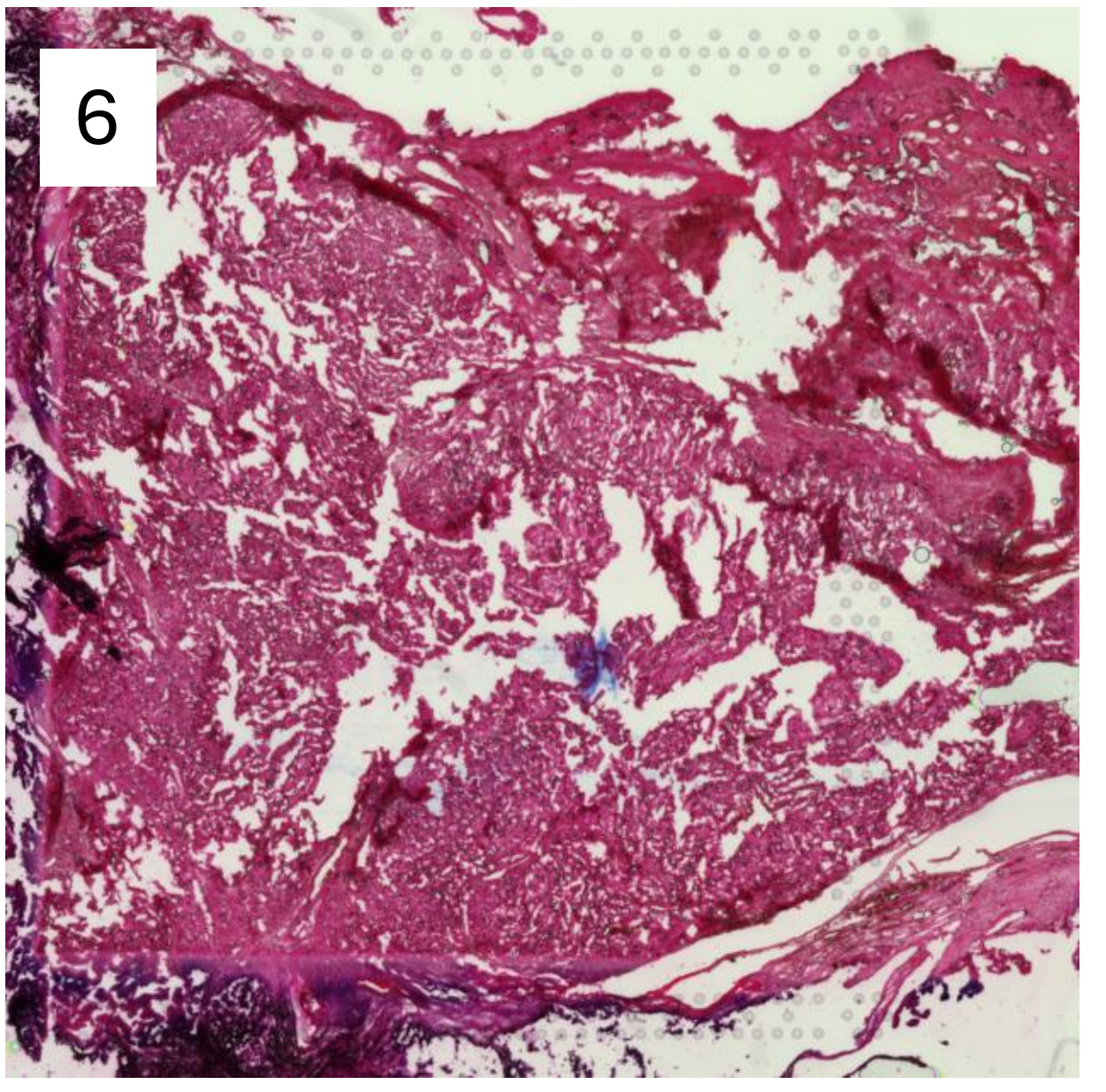
6
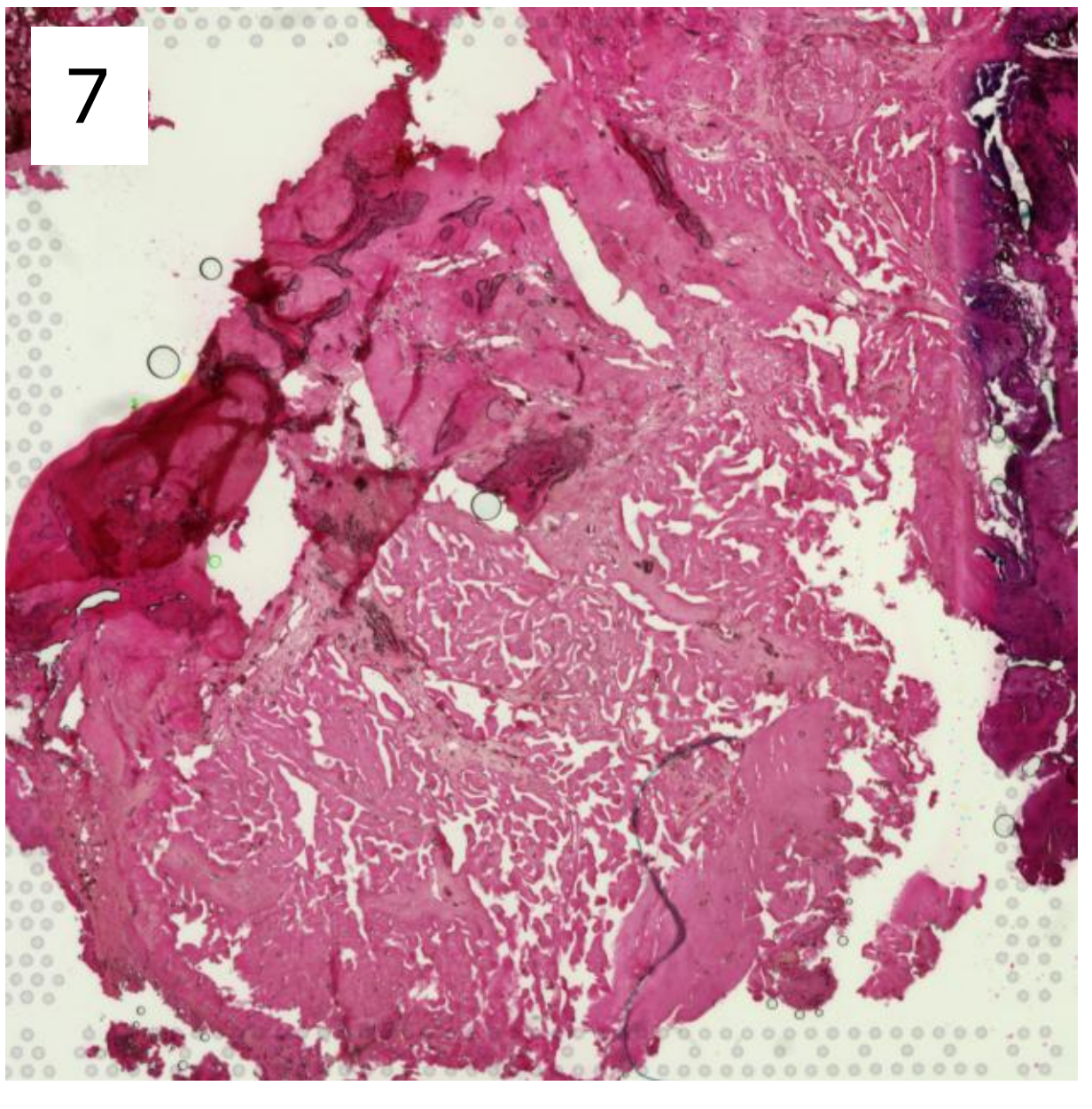
7
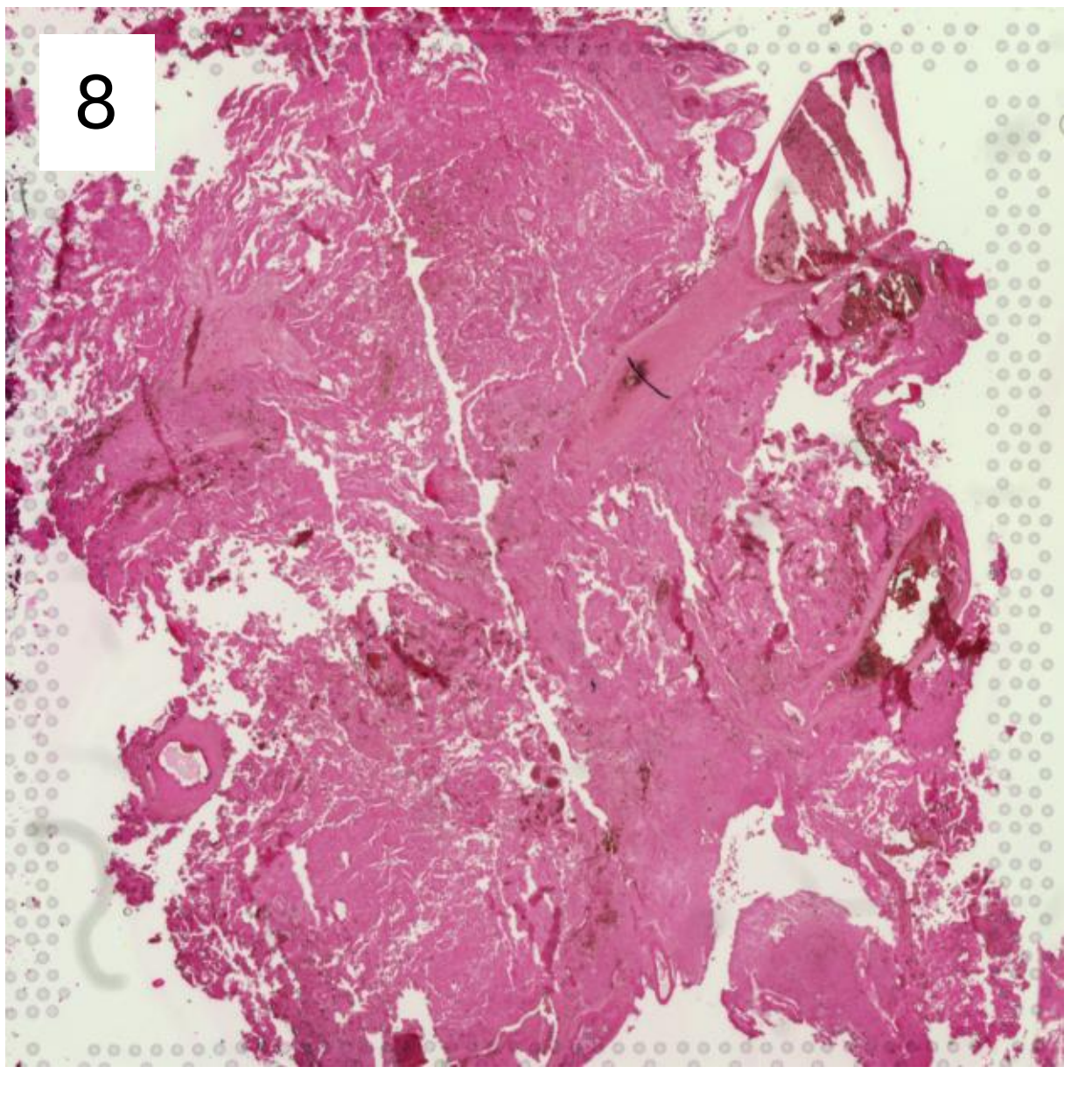
8
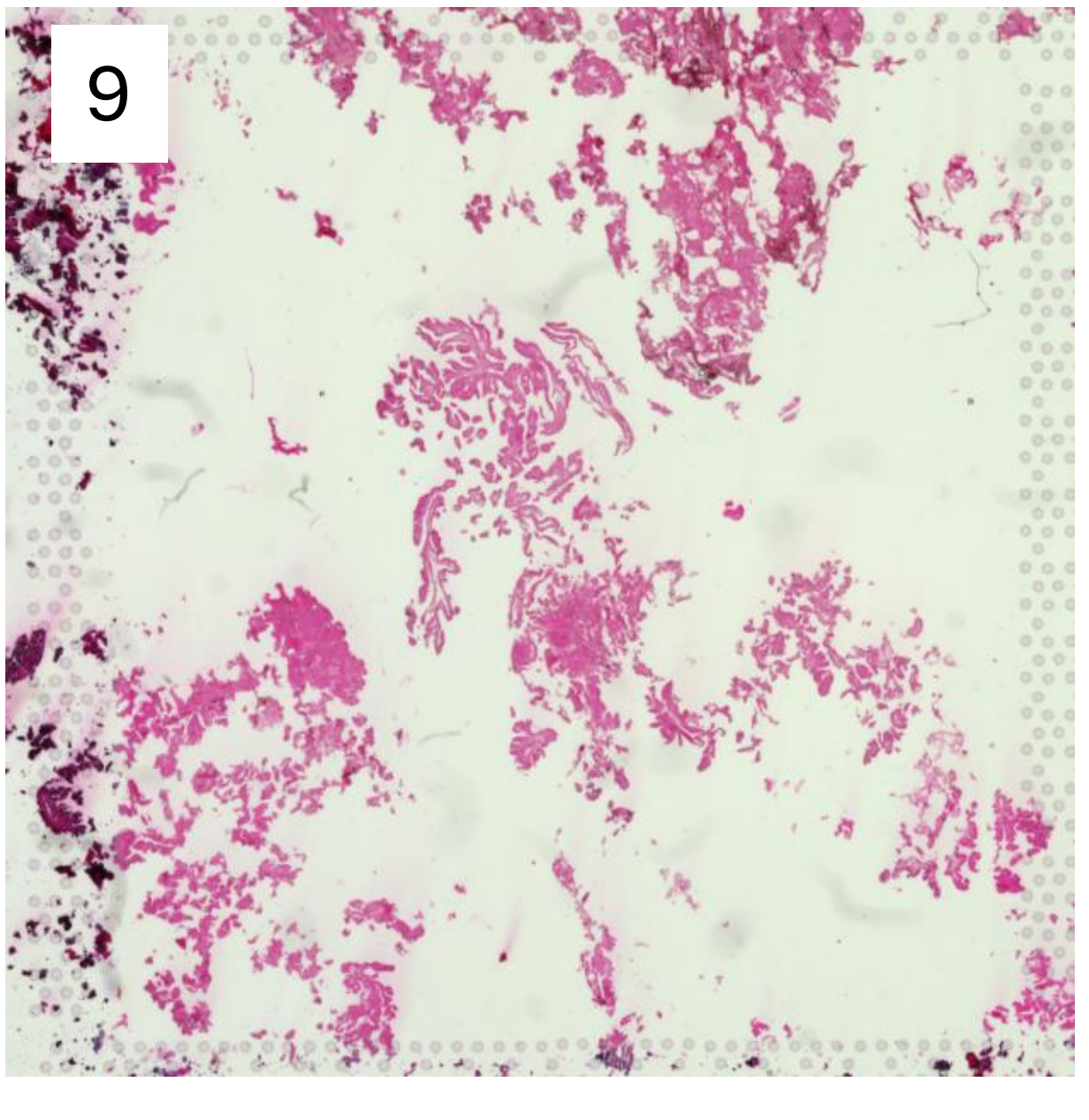
9
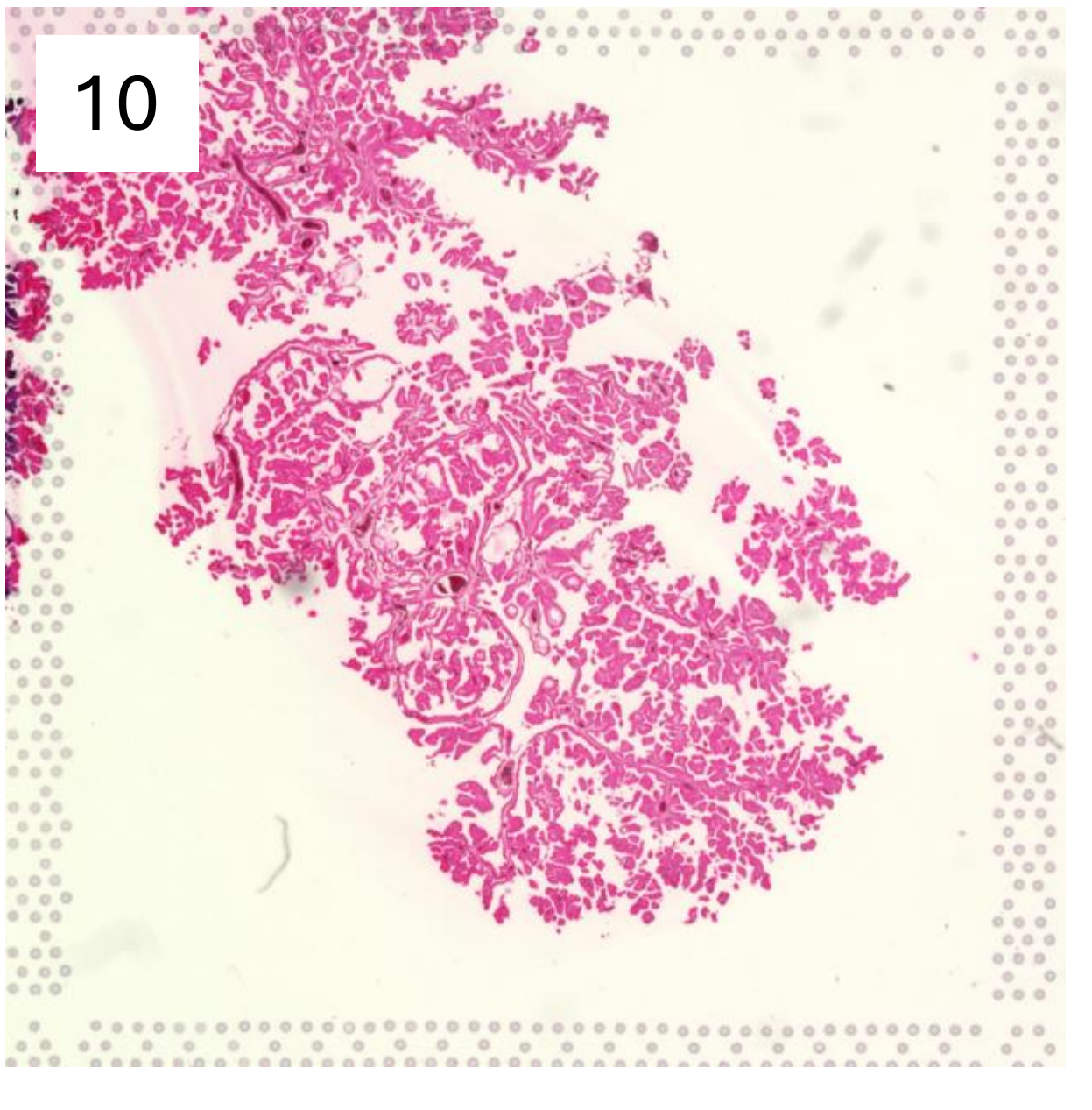
10

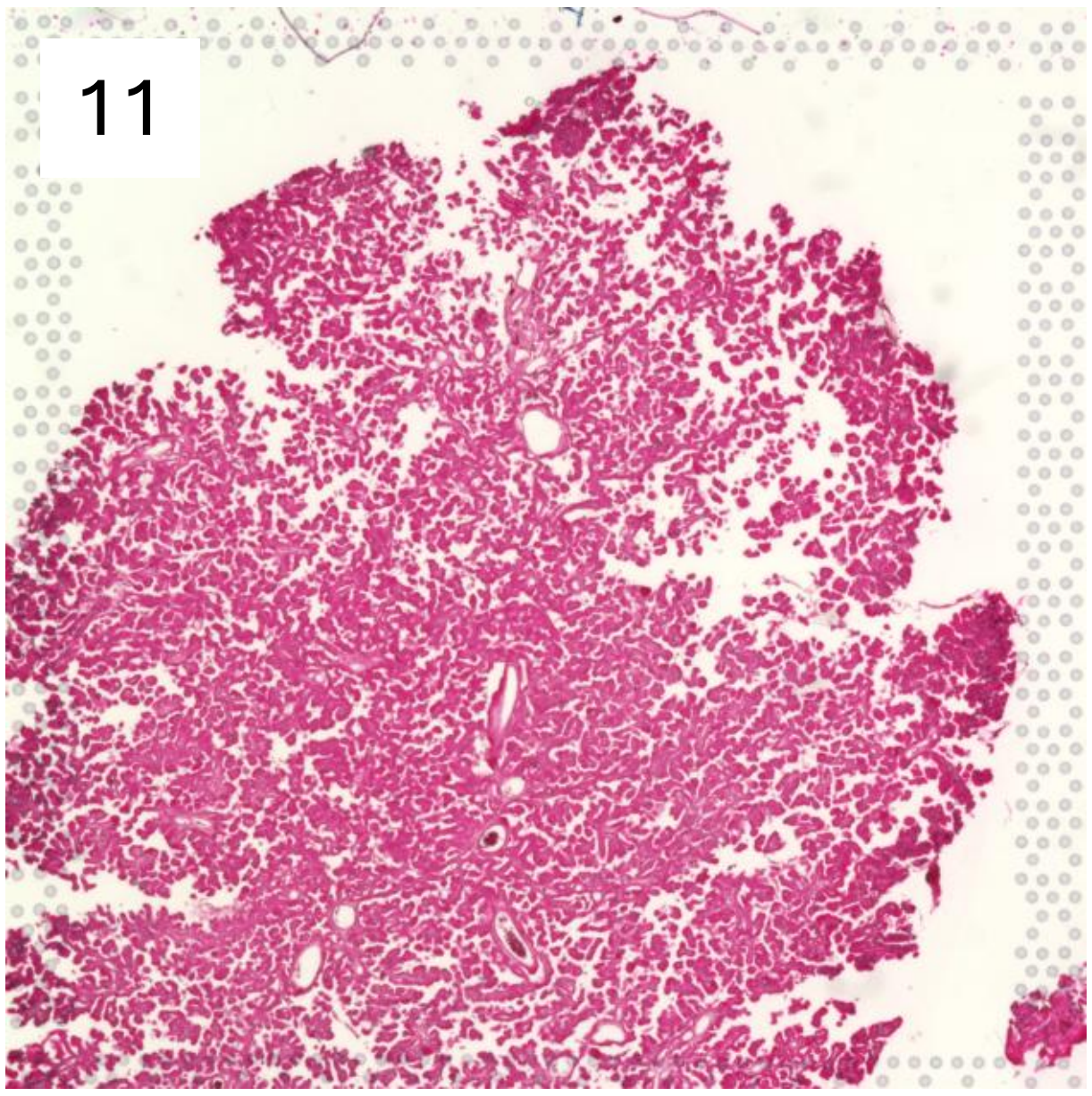
11

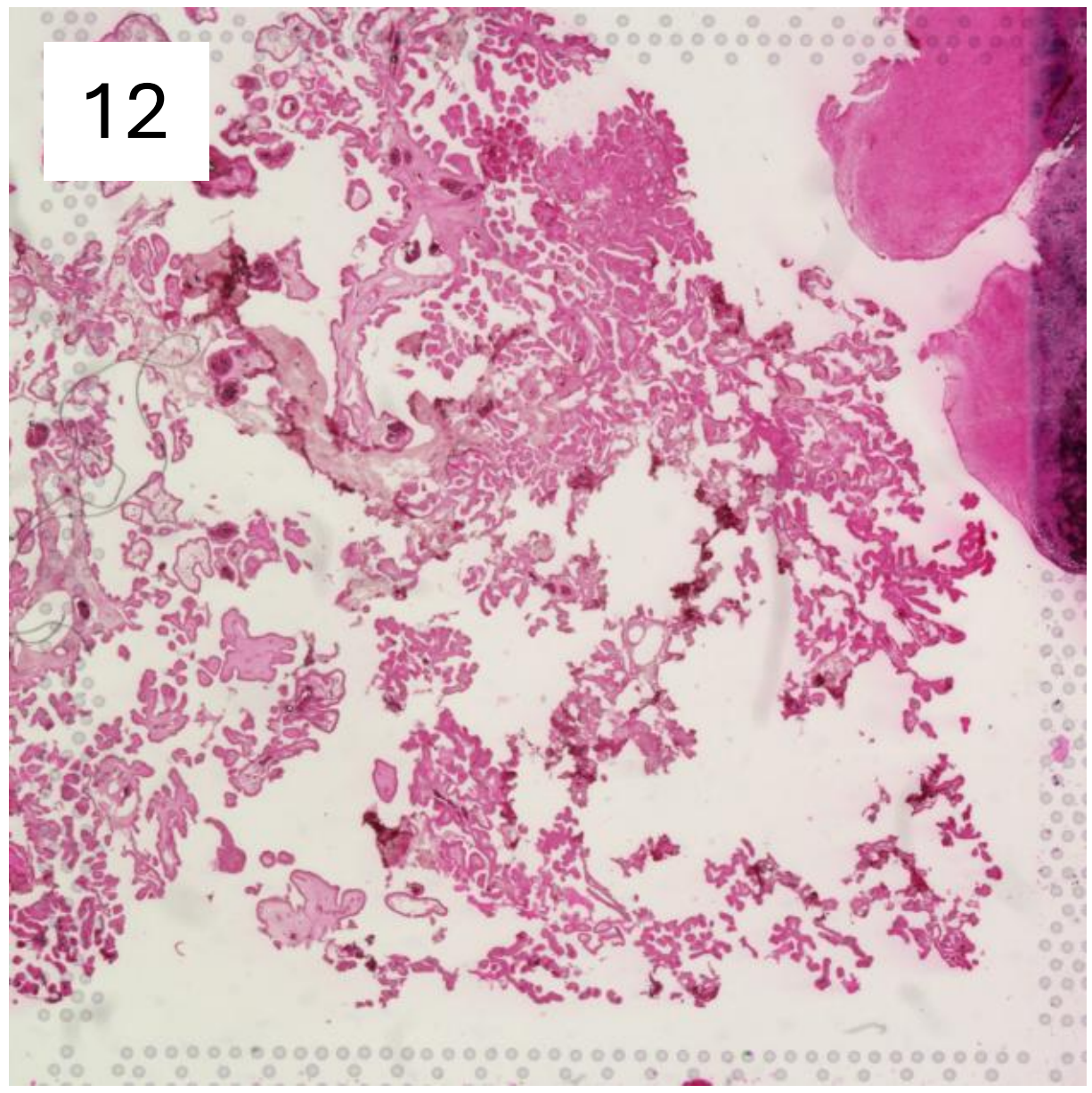
12

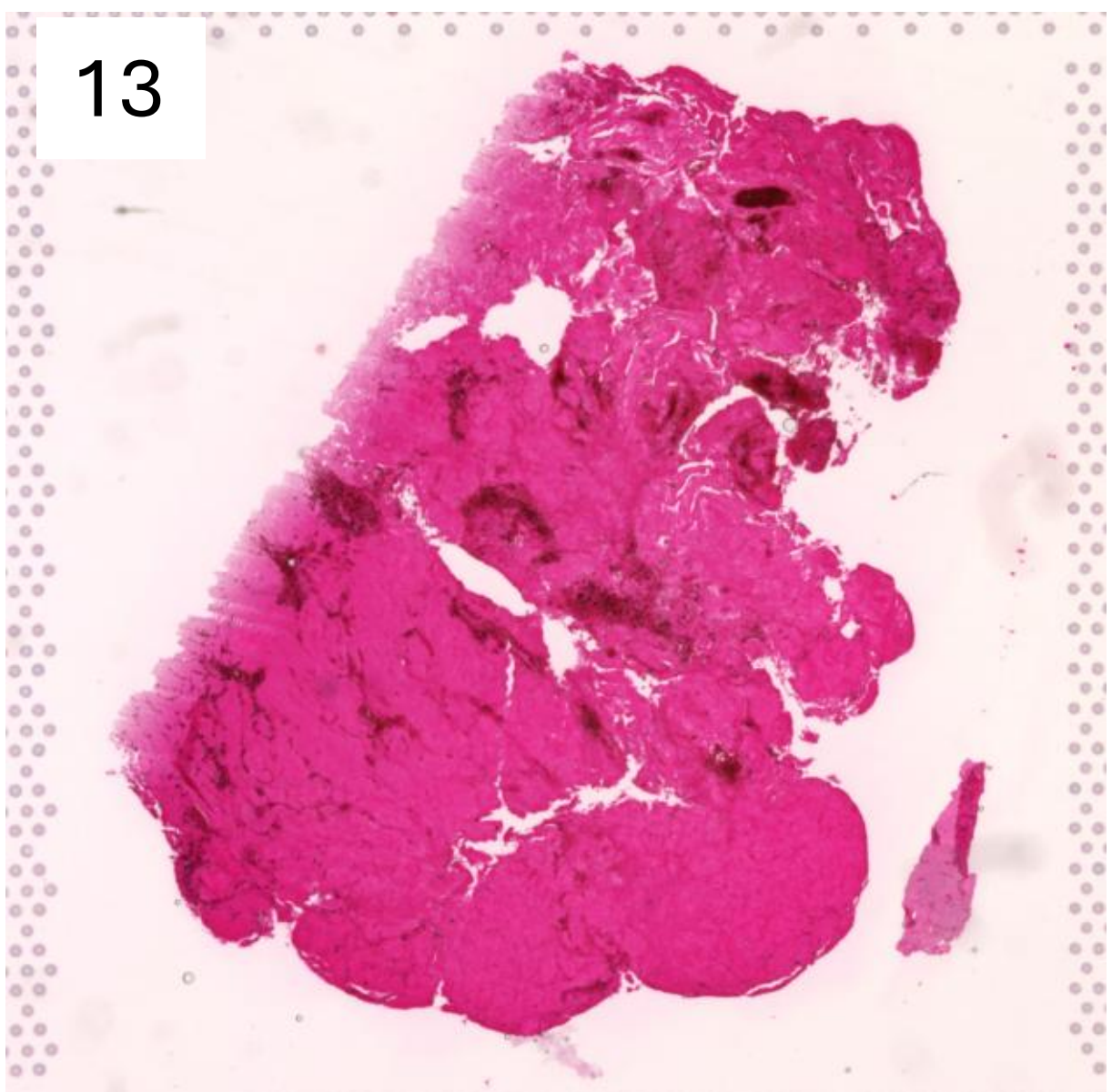
13

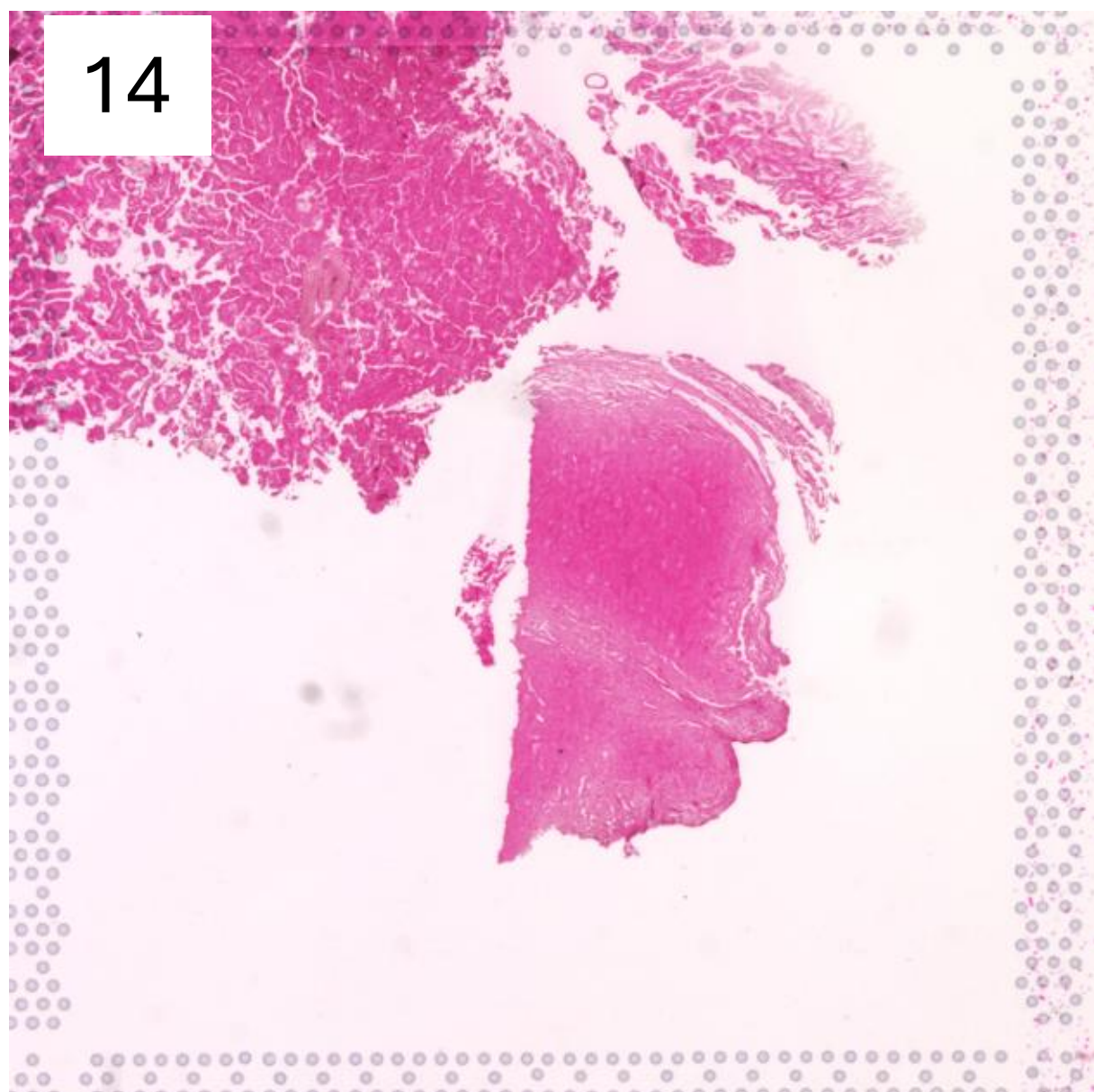
14

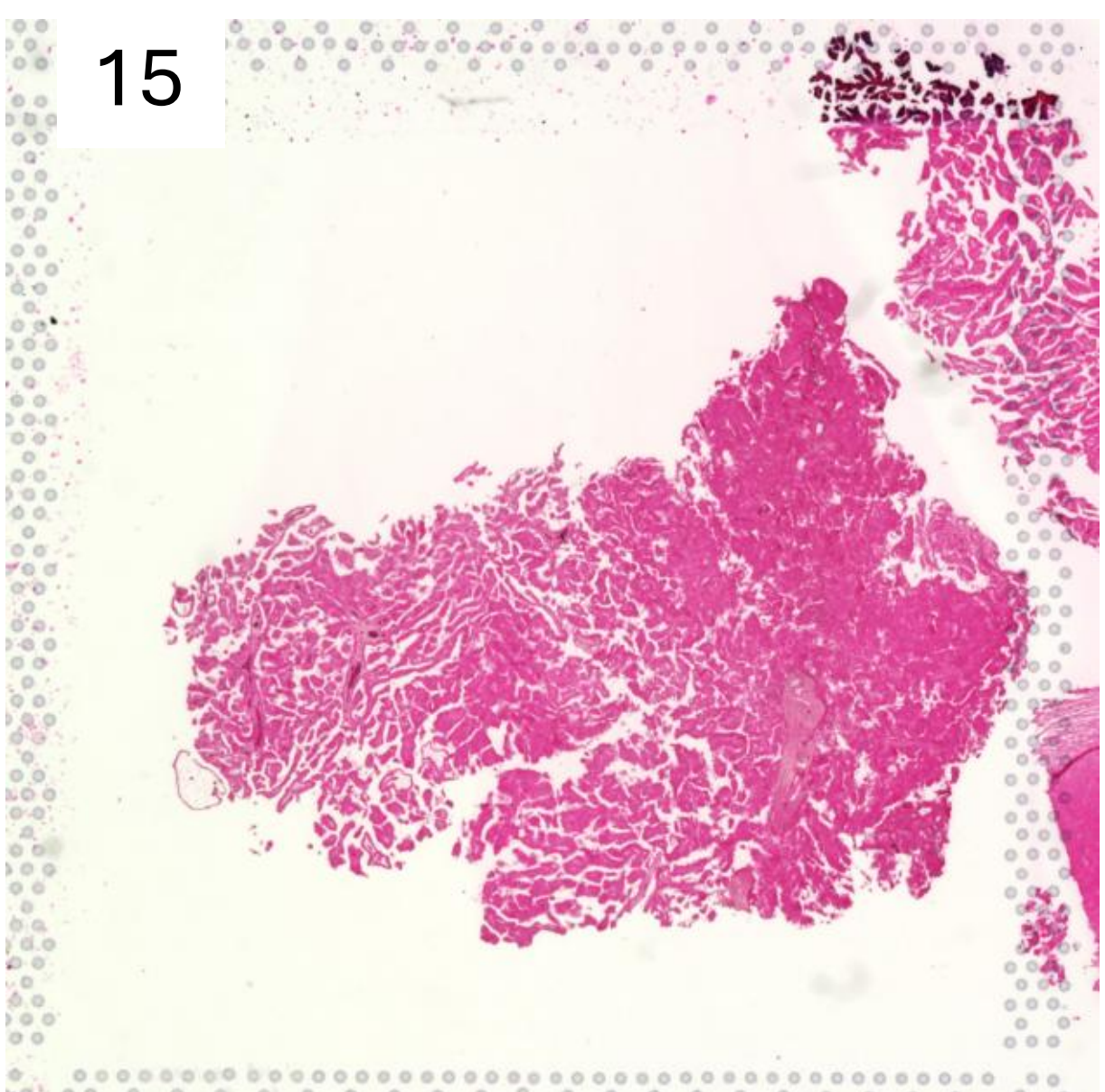
15

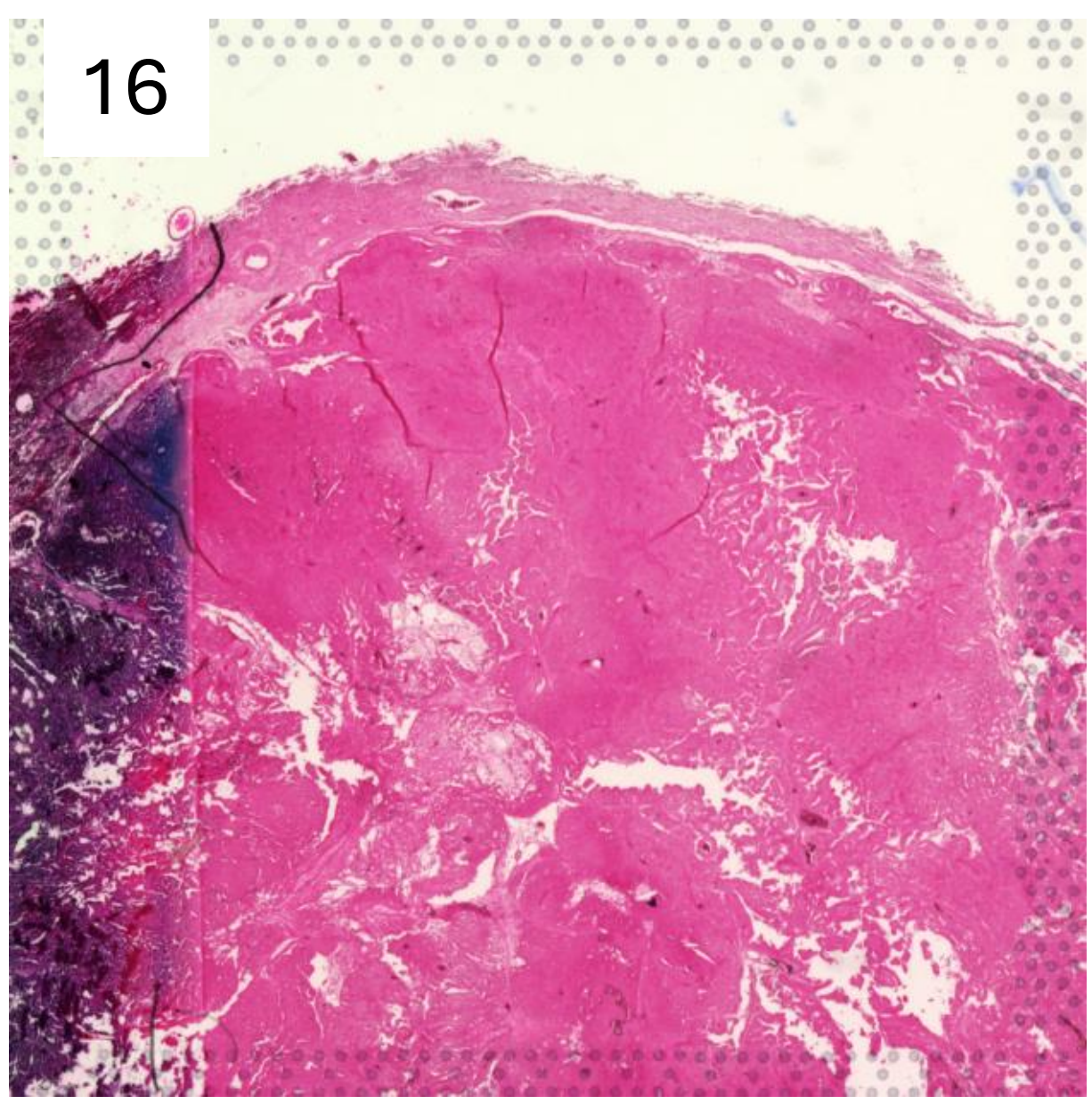
16

17

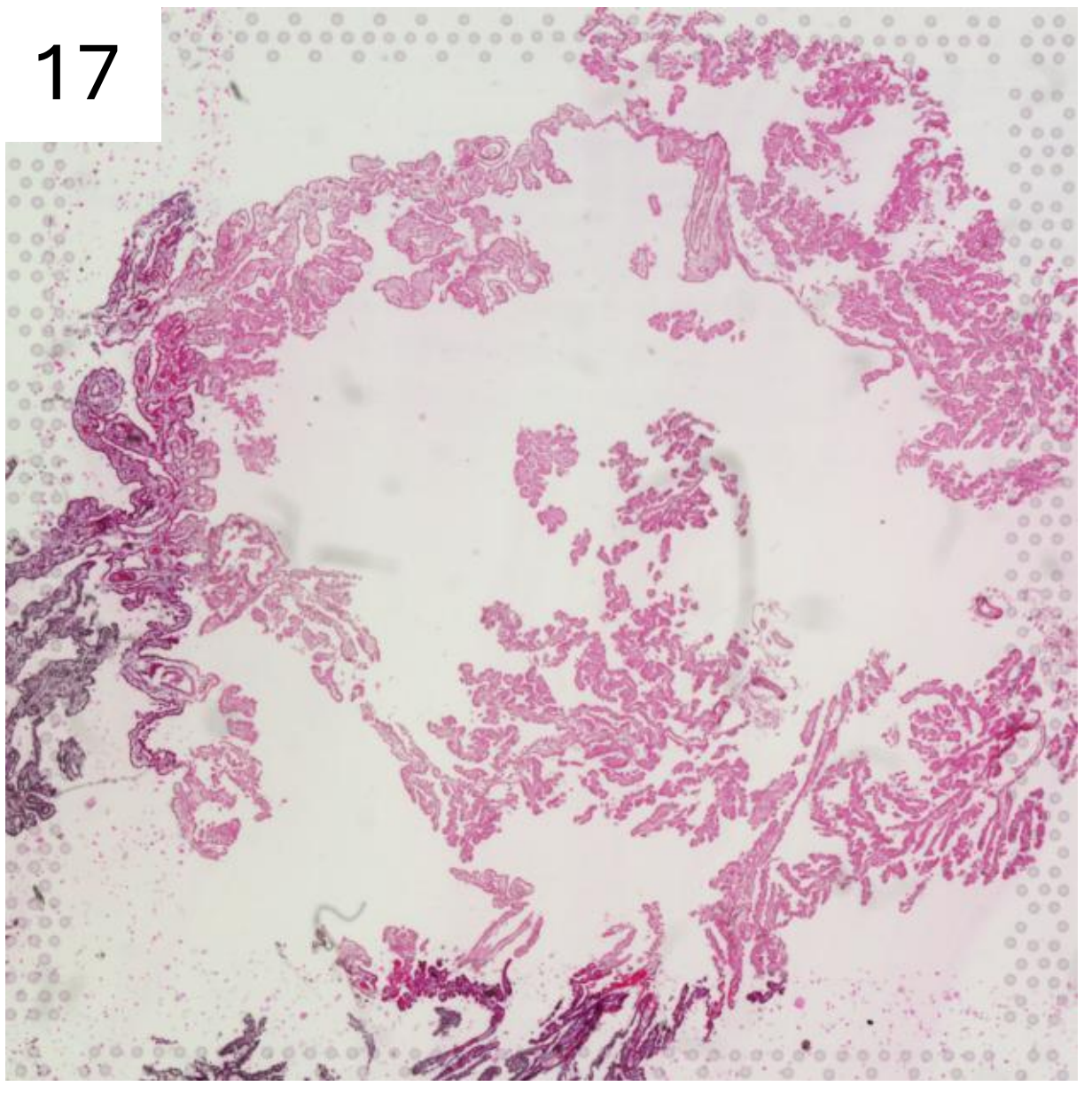

18

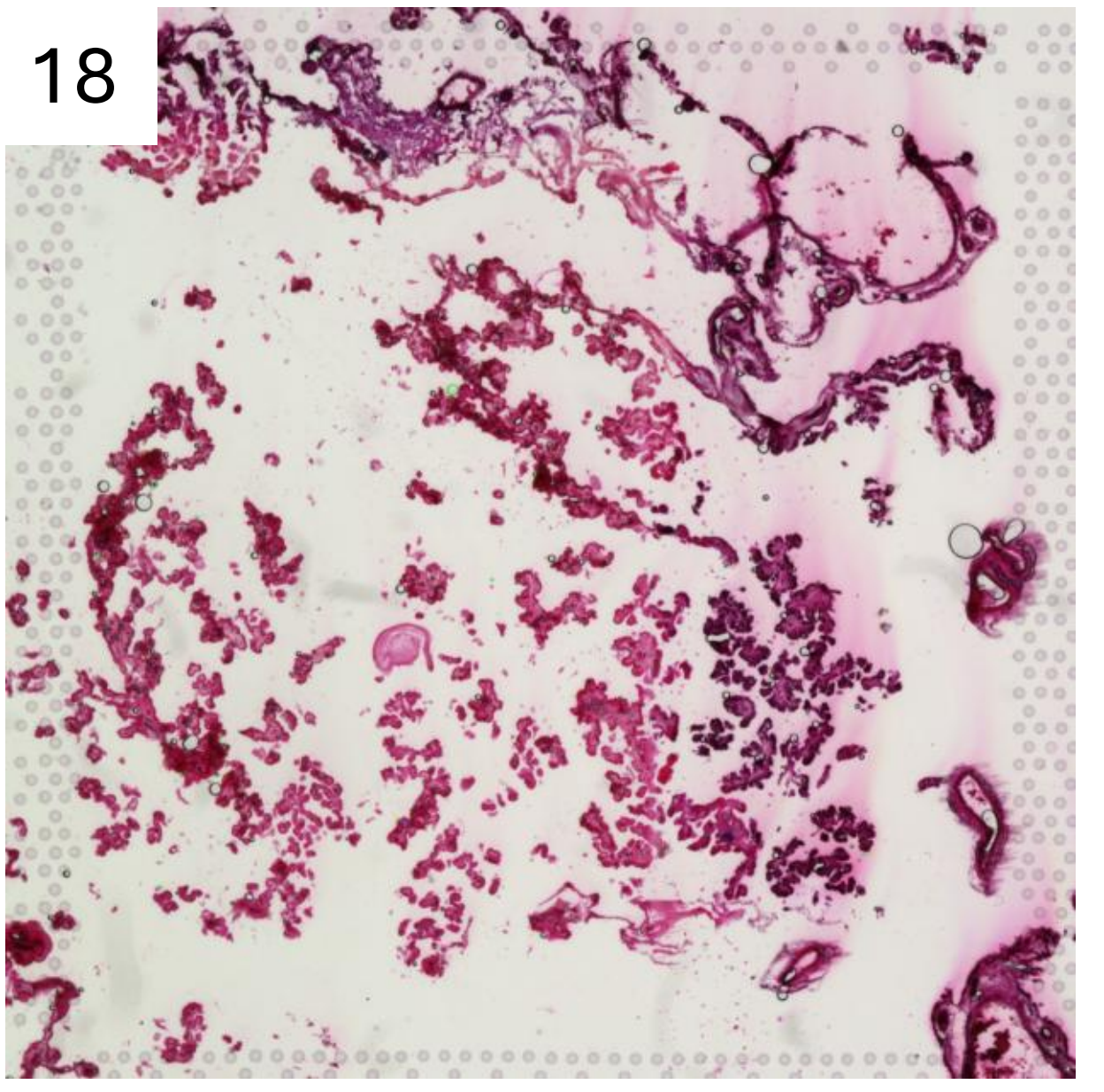

Supplement: Supplementary file 1 [file 13073_2026_1814_MOESM2_ESM.pdf]
