# Supplementary material for: Integrated single-nucleus and spatial transcriptomics define tumour cell metaprograms and tumour microenvironment organisation in choroid plexus tumours
Source: Genome Med. 2026 Nov 3;18:172. doi: 10.1186/s13073-026-01814-8 (PMC13737775; doi:10.1186/s13073-026-01814-8)
Supplement: Supplementary file 2 [file 13073_2026_1814_MOESM1_ESM.docx]

**Additional file 1: Supplementary Methods:**

**Human subject acquisition**

Formalin-fixed paraffin-embedded (FFPE) tumour blocks were collected from patients of University Hospital Münster (Germany), Hospital Recklinghausen (Germany), University Hospital Würzburg (Germany), Hospital Bremen (Germany), University Hospital Berlin (Germany), University Hospital Mainz (Germany), University Hospital Heidelberg (Germany), University Hospital Freiburg (Germany), University Hospital Gießen (Germany), University Hospital Regensburg (Germany), Hospital Hamm (Germany), University Hospital Dortmund (Germany), University Hospital Essen (Germany), University Hospital Leuven (Belgium,) Montreal (Canada), University Hospital Bonn (Germany) and Varese (Italy) with the consent of patients or their legal representatives. Criteria for initial collection of specimens were the histological diagnosis as Choroid Plexus Tumour WHO I, II or III and a clear assignment to one of the three methylation classes adult, pedA or pedB. Non-neoplastic, FFPE embedded ChP tissue of four foetal, three paediatric and five adult autopsy cases was collected from the archive of the Neuropathology in Münster.

**Table S1: Human tissue samples**

| Spatial | snRNA-seq | Relapse | Methylation | Sample | Sex | Age (y) |
| --- | --- | --- | --- | --- | --- | --- |
|  | X |  | adult | aCPP 01 | f | 63 |
| X | X |  | pedA | aCPP 02 | m | 0,17 |
|  | X |  | pedA | aCPP 03 | m | 0,17 |
|  | X |  | pedA | aCPP 04 | m | 0,67 |
|  | X |  | pedA | aCPP 05 | m | 1 |
|  | X |  | pedA | aCPP 06 | m | 11 |
|  | X |  | pedB | aCPP 07 | m | 6 |
|  | X | X | pedB | aCPP 08 | m | 6 |
|  | X |  | pedB | aCPP 09 | f | 1,33 |
|  | X |  | pedB | aCPP 10 | f | 12 |
| X (2) |  |  | pedB | aCPP 11 | m | 2,75 |
| X | X |  | pedB | CPC 01 | f | 0,23 |
|  | X |  | pedB | CPC 02 | m | 0,42 |
|  | X |  | pedB | CPC 03_p | f | 0,5 |
|  | X | x | pedB | CPC 03_r1 | f | 0,5 |
|  | X | x | pedB | CPC 03_r2 | f | 0,5 |
|  | X |  | pedB | CPC 04 | f | 1 |
| X (2) | X |  | pedB | CPC 05_p | m | 1 |
|  | X | x | pedB | CPC 05_r | m | 1 |
|  | X |  | pedB | CPC 06_p | f | 3 |
|  | X | x | pedB | CPC 06_r | f | 3 |
|  | X |  | pedB | CPC 07 | m | 11 |
| X | X |  | pedB | CPC 08 | m | 16 |
|  | X |  | pedB | CPC 09 | m | 27 |
|  | X |  | pedB | CPC 10 | m | 30 |
|  | X |  | pedB | CPC 11 | n/a | n/a |
|  | X |  | pedB | CPC 12 | m | 6,5 |
| X |  | X | pedB | CPC 13 | f | 16 |
| X | X |  | adult | CPP 01 | m | 6 |
|  | X |  | adult | CPP 02 | m | 15 |
|  | X |  | adult | CPP 03 | f | 18 |
|  | X |  | adult | CPP 04 | f | 23 |
| X | X |  | adult | CPP 05 | f | 33 |
| X | X |  | adult | CPP 06 | m | 43 |
|  | X |  | adult | CPP 07 | m | 45 |
|  | X |  | adult | CPP 08 | m | 46 |
| X | X |  | adult | CPP 09 | f | 52 |
|  | X |  | adult | CPP 10 | m | 56 |
| X | X |  | adult | CPP 11 | m | 58 |
|  | X |  | adult | CPP 12 | m | 72 |
|  | X |  | pedA | CPP 13 | m | 2 |
|  | X |  | pedA | CPP 14 | m | 3 |
| X | X | x (aCPP) | pedA | CPP 15 | m | 3 |
| X | X |  | adult | CPP 16 | f | 3,4 |
| X | X |  | pedB | CPP 17 | m | 0,9 |
|  | X |  | foetal | Fet 01 | n/a | GW22 |
|  | X |  | foetal | Fet 02 | n/a | GW28 |
|  | X |  | foetal | Fet 03 | n/a | GW29+2 |
|  | X |  | foetal | Fet 04 | n/a | GW34 |
| X | X |  | nn_pediatric | ChP 01 | m | 0,42 |
|  | X |  | nn_pediatric | ChP 02 | m | 0,5 |
|  | X |  | nn_pediatric | ChP 03 | m | 1,33 |
|  | X |  | nn_adult | ChP 04 | f | 20 |
|  | X |  | nn_adult | ChP 05 | f | 30 |
|  | X |  | nn_adult | ChP 06 | f | 34 |
|  | X |  | nn_adult | ChP 07 | m | 38 |
| X | X |  | nn_adult | ChP 08 | f | 46 |

**Primary processing of tumour samples**

**Sample selection and validation**. To confirm accurate determination of methylation classes, bulk methylation profiles (450k or EPIC arrays) were either generated de novo or obtained for all tumour specimens. DNA was isolated from FFPE material using the ReliaPrep™ FFPE gDNA Miniprep System (Promega) following standard protocols. For downstream analysis, 100–500 ng of DNA underwent bisulfite treatment with the EZ DNA Methylation Kit (Zymo Research). The converted DNA was then purified and restored using the DNA Clean & Concentrator-5 (Zymo Research) in combination with the Infinium HD FFPE DNA Restore Kit (Illumina). Subsequent methylation profiling was carried out with the Infinium MethylationEPIC BeadChip (Illumina), and signal detection was performed on an iScan platform (Illumina). Sample classification was performed with the Heidelberg Epignostix CNS Tumour Classifier v12.8 (https://epignostix.com/)^1^. Using this approach, 23 samples were confidently assigned to the pedB methylation subgroup, 8 to the pedA methylation subgroup and 14 to the adult subgroup (defined by a calibrated score > 0.8). Molecular subclasses were inferred either from confident methylation-based classifier results and/or from detection of fusion transcripts. Seven patients were excluded due to divergent or uncertain tumour classification, yielding a final cohort of 38 patients and a total of 45 specimens (Fig. 1a, b).

**Sample preparation for** **single-nucleus RNA sequencing**. For snRNA-seq a total of 43 CPT specimens (CPP: n = 17; aCPP: n = 10; CPC: n = 16) and additionally 12 non-neoplastic samples of choroid plexus (normal ChP: n = 8; foetal ChP: n = 4) were processed using the Single Cell Gene Expression Flex Fixed RNA Profiling workflow (10x Genomics, CG000632 Rev B). From each paraffin block, two to three 25 µm thick tissue sections were obtained. Sections were deparaffinised in xylene, rehydrated through graded ethanol, and enzymatically digested with Liberase TH (MilliporeSigma) on the gentleMACS Octo Dissociator with Heaters (Miltenyi Biotec, #130-096-427; program 37C_FFPE_1). After dissociation, nuclei were washed in PBS, passed through 30 µm strainers (Miltenyi Biotec), and transferred into quenching buffer (10x Genomics). The resulting suspensions were preserved in long-term storage solution containing 10% Enhancer (10x Genomics) and 10% glycerol at –20 °C. Prior to library construction, frozen suspensions were thawed, rinsed with PBS, re-filtered, and counted on a Countess 3 FL Automated Cell Counter (Invitrogen) using propidium iodide staining (ViaStain PI Staining Solution, Nexcelom). Multiplexed library preparation was carried out for 16 samples simultaneously, each assigned a unique barcode, according to the manufacturer’s protocol (10x Genomics, CG000527 Rev E). For probe hybridization, 5 × 10⁵ to 1 × 10⁶ nuclei per sample were used and incubated with genome-wide probe panels (10x Genomics). After hybridization, nuclei were washed, pooled in groups of 16, and loaded onto a Chromium iX chip targeting a recovery of 8,000 nuclei per pool. Inside the GEMs, ligation of probes and GEM barcoding were performed, followed by pre-amplification to generate sequencing-ready libraries. The final libraries were sequenced on an Illumina NovaSeq 6000 platform using a 200-cycle, paired-end high-throughput kit.

**Sample preparation for spatial transcriptomics.** Tissue sections from 16 CPT and 2 normal ChP FFPE tissue blocks were processed by Visium CytAssist Spatial Gene Expression for FFPE v2 using Visium V4 slides for mRNA capture. Prior to the H&E imaging performed as part of the protocol, bright-field images of adjacent H&E-stained sections were captured using a Vectra 3 system (Akoya Biosciences) with 4x or 10x objectives to help delineate regions of interest. Processed tissue sections were subsequently scanned on a Grundium Ocus40 digital slide scanner (Grundium Ltd) with a 20x objective. The full resolution images of the whole-slide images are available via OMERO (<https://omero-imaging.uni-muenster.de/webclient/img_detail/761636/>). Library concentration and fragment size distributions were determined on a TapeStation 2000 (Agilent Technologies), and sequencing was carried out on the Illumina NextSeq 2000 platform using a high-throughput kit (100 cycles, paired-end).

**Statistical analysis of methylation, single cell and spatial data**

**Quality control, preprocessing and normalization**. Analysis of snRNA-seq data was performed in R (version 4.4.0)^2^, using the package Seurat (version 5.3.0)^3,4^. All the datasets were loaded in R separately, retaining genes expressed in at least 3 cells, and cells expressing at least 50 genes. After assessing the distributions of number of genes and percentage of mitochondrial genes (percent.mito) by number of UMI counts per sample, we determined filtering cutoffs equal for all samples. In particular, we excluded cells with a number of genes <200 and percent.mito > 25%. We chose this permissive threshold in order not to filter out tumour cells which may have a higher mitochondrial gene expression for biological reasons, rather than being apoptotic cells. The number of nuclei per sample was limited to 5,000 to reduce numerical imbalance before merging the count data of all individual samples. Normalization was performed using the Seurat function NormalizeData (method = “LogNormalize”, scale.factor = 10,000). Finally, we calculated highly variable genes (n = 2000) with the selection method “vst”.

**Sample integration, UMAP, clustering and annotation for whole samples and tumour subset.** To reduce technical intersample variance introduced by batch effects in integrated downstream analyses while conserving biological heterogeneity, RPCA-Integration^5^ of all tumour and non-neoplastic samples was performed with 20 integration anchors on the 8 technical batches in which the samples were processed. Batch correction was performed at the level of technical sequencing batches, corresponding to independent library preparation and sequencing runs, and not at the level of individual tumour samples. This strategy was chosen to correct technical variation introduced during sample processing and sequencing while avoiding overcorrection of biologically meaningful inter-patient differences. Louvain clustering^6^ with a resolution of 0.5 and uniform manifold approximation and projection (UMAP)^7^ were performed based on the top 30 principal components for functional separation and dimensionality reduction of the cells. The resulting 26 clusters were annotated based on established cell type-specific marker genes^8,9^. Clusters representing the same cellular identity were subsequently merged, resulting in eight major cell populations (Fig. 1c). One cluster was characterised by high expression of mitochondrial genes together with genes associated with cellular stress, consistent with necrotic or damaged nuclei. This cluster was retained during the initial clustering and annotation steps to provide a complete overview of the cellular composition but was excluded from downstream analyses of the tumour cell subset to avoid potential technical bias.

For analysis of the intertumoural heterogeneity, all tumour samples were subsetted from the whole Seurat object, repeating Louvain clustering and UMAP dimension reduction. Differentially expressed genes (DEG) between a cluster and all other clusters were identified using FindAllMarkers() with a minimal log2 fold change (FC) of 0.25. In addition to canonical marker genes from a recently published murine single-cell atlas^12^, clusterProfiler version 4.12.6 (RRID:SCR_016884)^10^ was used to annotate clusters with three collections of reference gene sets from the human MSigDB^11^ (C8, C2 (subcategory = "CP:WIKIPATHWAYS”, “CP:REACTOME), and C5), covering broad human cell types, canonical pathways, and Gene Ontology signatures.

**Identification of tumour consensus programs by non-negative matrix factorisation.**
To uncover shared transcriptional programs, we applied non-negative matrix factorisation (NMF) independently to each tumour sample. The analysis was restricted to malignant nuclei and based on the scaled expression of the 3,000 most variable genes, testing ranks from 2 to 15. For every sample, this procedure generated multiple transcriptional programs (here referred to as “sample-programs”), which were represented by the 50 genes with the highest NMF loadings. Following the strategies proposed by Gavish *et al*.^12^ and Muenter *et al*.^13^, we derived consensus programs (or metaprograms) by identifying robust sample-programs and clustering them according to shared gene content. Robust sample-programs were defined as those from all ranks up to and including the highest rank for which the number of programs equalled the rank itself. To manage the high program count, we introduced relatively strict thresholds for robustness, non-redundancy within a given sample, and similarity across samples (set to 35, 10, and 10, respectively). This filtering yielded 239 robust sample-programs. For clustering, robust sample-programs were merged into consensus programs if they shared at least 10 genes, and a minimum of 3 similar robust sample-programs was required to initiate a consensus program. To assess the dominance of each program within individual cells, we evaluated the nuclei based on the expression of their respective 50 constituent genes. For functional annotation, pathway analyses were conducted using the complete list of constituent genes for each metaprogram. Labels were assigned based on the predominant GO-terms (Biological Process (BP), Molecular Function (MF) and Cellular Component (CC)) consistently identified with a combination of tools including clusterProfiler (v.4.20.0)^10^ and ToppGene webbrowser^14^.

**Longitudinal analysis of two CPC patients with different clinical outcomes:** Dynamic modulation of these metaprograms was evident in two paediatric CPC patients for whom matched pre- and post-chemotherapy specimens were included in the cohort (CPC_05 and CPC_03). Patient 1 (CPC_03) exhibited an excellent clinical course, with no more sign of malignancy after treatment and histological regression (formally to WHO grade 2) in a second-look operation 14 months after initial tumour resection and long-term remission following a watch-and-wait strategy (follow-up time: 165 months). In contrast, patient 2 (CPC_05) experienced a fulminant course with tumour re-resections after 13 months and subsequent metastatic spread into the fourth ventricle after 22 months. In line with these clinical observations, stress-response and TGF-β signalling and to a lesser extent hypoxia and interferon programs were increased after therapy in patient 2, whereas these signatures were unaltered or reduced following treatment in patient 1.

**Spatial transcriptomic data processing and integration with snRNA‑seq.** Raw FASTQ files from Visium FFPE libraries were processed with the 10x Genomics Space Ranger pipeline (version 2.1.0)^15^ using the “count” pipeline and the GRCh38-2020-A human reference with otherwise default settings. Manual (re)alignment of fiducials was performed for samples aCPP 02, aCPP 11 (2x), CPP 01, CPC 01, CPP 05, CPC 05_p (2x), CPC 13, CPP 15, CPP 17, CPP 16, CPP 09, CPP 11, CPP 06, CPC 08, ChP 01, ChP 08 using Loupe Browser version 8.0.0 (10x Genomics, RRID:SCR_018555)^16^. Count data were loaded into R (version 4.4.0) using Seurat’s (Seurat version 5.3.0)^3,4^ Load10X_Spatial() and for each Visium capture area, we retained spots with at least 1000 detected genes (nFeats) and 10,000 unique molecular identifiers (UMIs) (nCounts), and excluded spots with a mitochondrial RNA fraction >25%. To relate spatial spots to tumour and non‑tumour compartments, we first restricted the analysis to spots within the H&E‑annotated tissue mask and, where applicable, to regions of interest (ROIs) manually delineated by a neuropathologist. We then computed module scores for gene sets corresponding to the ten single‑nucleus‑derived metaprograms (MP1–MP10) and selected lineage or stromal signatures using Seurat’s AddModuleScore function with default parameters. For visualisation, module scores were rescaled to the [0, 1] interval per slide. Spatial co‑enrichment and anticorrelation between metaprograms (e.g. hypoxia vs ciliogenesis) were quantified by computing spot‑wise Pearson correlation coefficients between module scores across all spots within a given section. For summary analyses, correlation coefficients were averaged across samples within the same methylation subgroup. Spatial feature plots and spot‑level heatmaps were generated with Seurat (SpatialFeaturePlot, DoHeatmap) and custom ggplot2^17^ code.

**Myeloid subclustering and TAM state annotation.** For myeloid re‑clustering, we subsetted all clusters from the integrated object of all tumour samples that were annotated as myeloid cells based on expression of canonical markers (e.g. *C1QC*, *CD14*, *CD68*, *CD163*, *CX3CR1*) and the original clustering labels. The myeloid subset was normalised and scaled using Seurat (NormalizeData, ScaleData), and highly variable genes 2,000 were identified with the “vst” method. Dimensionality reduction was performed by principal component analysis on the variable genes, and the first 30 principal components were used to construct a shared nearest neighbour graph (FindNeighbours, k.param = 30). Unsupervised clustering was carried out with the Louvain algorithm^6^ using FindClusters (resolution = 0.4), yielding nine myeloid clusters. Former cluster 4 contained mixed gene expression of myeloid genes and tumour/epithelial markers and was therefore excluded from annotation and analysis. UMAP embeddings were generated using RunUMAP with default parameters for visualisation. TAM states were assigned to clusters using a combination of marker genes and reference signatures from published TAM atlases^18^. Clusters with high expression of *TREM2*, *APOE*, *SPP1*, *TMEM119*, *CX3CR1* were annotated as *TREM2*⁺ / microglia‑like TAMs; clusters enriched for *LYVE1*, *FOLR2* and *MRC1* were annotated as resident tissue macrophage‑like TAMs; clusters characterised by *ISG15, IFIT1, IFI44L* and *CXCL10* were designated interferon‑responsive TAMs (IFN‑TAMs); and clusters expressing *APOC1, APOC2, FABP5* and *LGALS3* were classified as lipid‑associated TAMs. Additional clusters with high *VCAN* and *S100A8/9* were annotated as tumour‑infiltrating monocytes, inflammatory TAMs with expression of chemokines such as *CCL2*, *CCL3L1* and *EGR3* and proliferating TAMs, expressing *MKI67*, *TOP2A* and *CDK1*.

Differential expression between pedB‑derived TAMs and TAMs from other subgroups was computed using Seurat’s FindMarkers function (test = MAST^19^, min.pct = 0.2). P values were adjusted for multiple testing using the Benjamini–Hochberg method, and genes with adjusted P < 0.05 and log₂ fold change > 0.25 were considered significant. Gene Ontology and pathway enrichment analyses of TAM‑specific signatures were performed using clusterProfiler^10^ webbrowser and ToppGene^14^ with default settings.

**Ligand–receptor analysis.** To infer intercellular communication between tumour cells, TAM subsets and stromal populations, we used the CellChat framework (CellChat version [2.0.0])^20,21^ in R. For this analysis, we constructed CellChat objects from the snRNA‑seq data using the normalised expression matrix and the cell type labels (tumour epithelium metaprograms, TAM subtypes, endothelial cells, pericytes, fibroblasts, lymphocytes and astroglial-like). We employed the “CellChatDB.human” ligand–receptor database and retained interactions with a minimum expression fraction of 0.1 in the sending and receiving populations and a computed communication probability above 0.05 (default settings). For pathway‑level analyses, we grouped ligand–receptor pairs into curated signalling pathways (e.g. *TGF‑β*, *VEGF*, *CSF*, chemokine pathways) using the built‑in CellChat annotations. To integrate ligand–receptor predictions with spatial information, we overlaid CellChat‑derived sender and receiver populations onto the corresponding Visium sections via their metaprogram and module scores, and qualitatively assessed whether predicted strong interactions (e.g. *TGF‑β* from MP7 tumour cells to *LYVE1*⁺ TAMs; *VEGF* from hypoxic tumour cells to endothelium) occurred in spatially co‑localised regions.

**Bulk RNA‑seq validation, digital cytometry and survival analysis.** For independent validation, we used a published bulk RNA‑seq dataset of 50 CPTs spanning all histological and methylation subgroups (CPP, aCPP, CPC; pedA, pedB, adult)^22^. DESeq2-normalised^23^ counts were provided by one of the original authors C.T. and imported into R. The DESeq2-normalised counts and the sample sheet of this cohort are provided in Additional file 4 (Tables S4 and S5). Clinical data was collected for 34 samples. Bulk metaprogram scores were computed by averaging the normalised scaled expression of the 50 hallmark genes defining each NMF‑derived metaprogram (MP1–MP10) or specific functional gene sets identified by GO Analysis (e.g. TGFβ-signalling response, Interferon I signaling) within each bulk sample / methylation group, after z‑scaling genes across samples. For some analyses, scores were further z‑normalised across the cohort. For survival analyses, samples were stratified into “high” and “low” expression groups using the R package maxstat^24^, which identifies the threshold that maximizes the separation between survival groups based on maximally selected rank statistics. Progression‑free survival (PFS) and overall survival (OS) were defined as time from diagnosis to progression/relapse or death, respectively, with censoring at last follow‑up. Digital cytometry of myeloid and TAM states from bulk RNA‑seq was performed using CIBERSORTx (https://cibersortx.stanford.edu/index.php)^25^. We constructed a custom signature matrix from the snRNA‑seq data by averaging expression profiles of the annotated major celltypes, MP annotations and myeloid clusters (same annotation as CellChat-Input) and submitted this matrix to CIBERSORTx using absolute mode, batch correction disabled, and 100 permutations. Quantile normalisation was disabled according to CIBERSORTx recommendations for RNA‑seq input. Estimated cell‑type fractions (or scores) were then correlated with methylation class and clinical variables and used as covariates in survival models. Kaplan-Meier curves and Cox proportional hazards models in the survival analyses were carried out in R using the survival^26^ and survminer^27^ packages (version 0.5.1). Kaplan–Meier curves were compared using log‑rank tests, and hazard ratios (HRs) with 95% confidence intervals (CIs) were estimated with Cox proportional hazards models. P values < 0.05 were considered statistically significant unless stated otherwise.

**Immunohistochemistry.** Immunohistochemical staining was performed for 19 CPT samples comprising four adult, five pedA and 10 pedB tumours (including seven CPCs) to validate selected transcriptomic findings at protein level using the following antibodies: anti-GT335 (AdipoGen Life Sciences, AG-20B-0020-C100, dilution 1:1,000, citrate pretreatment), anti-HIF1A (Abcam, clone H1alpha67, dilution 1:600, citrate buffer pH 6.0 pretreatment), and anti-Kir7.1 (kindly provided by S. Hirose, polyclonal rabbit anti-human, dilution 1:2,000, citrate pretreatment). All stainings were performed on a Dako Link48 Autostainer platform.

GT335 was used to assess ciliation and evaluated by quantitative microscopic assessment. The percentage of epithelial cells exhibiting mono- or multiciliation was recorded for each sample and quantified per subgroup (Additional file 2: Fig. S2d; Table S2). Kir7.1 immunostaining was performed as an orthogonal validation of the epithelial differentiation programme, as Kir7.1 is a potassium channel ubiquitously expressed in differentiated choroid plexus epithelial cells and involved in cerebrospinal fluid secretion. HIF1α immunohistochemistry was used to assess hypoxic tumour regions. Representative immunohistochemical images are shown in Additional file 2: Figs. S5–S7.

**Table S2: Quantification of mono- and multiciliated epithelial cells by GT335 immunohistochemistry**

|  | Diagnosis | DNA- methylation group | Monociliated epithelial cells (%) | Multiciliated epithelial cells (%) |
| --- | --- | --- | --- | --- |
| 1 | aCPP | adult | 70 | 20 |
| 2 | aCPP | adult | 20 | 60 |
| 3 | aCPP | pedA | 70 | 30 |
| 4 | CPP | pedA | 10 | 90 |
| 5 | aCPP | pedA | 0 | 100 |
| 6 | CPP | pedA | 50 | 40 |
| 7 | CPP | pedA | 20 | 80 |
| 8 | aCPP | pedB | 15 | 5 |
| 9 | CPC | pedB | 40 | 5 |
| 10 | CPC | pedB | 5 | 0 |
| 11 | aCPP | pedB | 50 | 20 |
| 12 | CPC | pedB | 25 | 0 |
| 13 | CPC | pedB | 0 | 0 |
| 14 | CPC | pedB | 5 | 0 |
| 15 | CPC | pedB | 0 | 0 |

**References**

1. Capper D, Jones DTW, Sill M, et al. DNA methylation-based classification of central nervous system tumours. Nature. 2018;555(7697):469-474. doi:10.1038/nature26000

2. R Core Team. R: A Language and Environment for Statistical Computing. R Foundation for Statistical Computing. 2024. <https://www.R-project.org/>

3. Hao Y, Stuart T, Kowalski MH, et al. Dictionary learning for integrative, multimodal and scalable single-cell analysis. Nat Biotechnol. 2024;42(2):293-304. doi:10.1038/s41587-023-01767-y

4. Stuart T, Butler A, Hoffman P, et al. Comprehensive Integration of Single-Cell Data. Cell. 2019;177(7):1888-1902.e21. doi:10.1016/j.cell.2019.05.031

5. Hao Y, Hao S, Andersen-Nissen E, et al. Integrated analysis of multimodal single-cell data. Cell. 2021;184(13):3573-3587.e29. doi:10.1016/j.cell.2021.04.048

6. Blondel VD, Guillaume JL, Lambiotte R, Lefebvre E. Fast unfolding of communities in large networks. J Stat Mech. 2008;2008(10):P10008. doi:10.1088/1742-5468/2008/10/P10008

7. McInnes L, Healy J, Saul N, Großberger L. UMAP: Uniform Manifold Approximation and Projection. J Open Source Softw. 2018;3(29):861. doi:10.21105/joss.00861

8. Dani N, Herbst RH, McCabe C, et al. A cellular and spatial map of the choroid plexus across brain ventricles and ages. Cell. 2021;184(11):3056-3074.e21. doi:10.1016/j.cell.2021.04.003

9. Hill AD, Okonechnikov K, Herr MK, et al. Single-nucleus RNA-seq dissection of choroid plexus tumor cell heterogeneity. EMBO J. 2024;43(24):6766-6791. doi:10.1038/s44318-024-00283-2

10. Wu T, Hu E, Xu S, et al. clusterProfiler 4.0: A universal enrichment tool for interpreting omics data. Innovation. 2021;2(3):100141. doi:10.1016/j.xinn.2021.100141

11. Liberzon A, Subramanian A, Pinchback R, Thorvaldsdóttir H, Tamayo P, Mesirov JP. Molecular signatures database (MSigDB) 3.0. Bioinformatics. 2011;27(12):1739-1740. doi:10.1093/bioinformatics/btr260

12. Gavish A, Tyler M, Greenwald AC, et al. Hallmarks of transcriptional intratumour heterogeneity across a thousand tumours. Nature. 2023;618(7965):598-606. doi:10.1038/s41586-023-06130-4

13. Münter D, De Faria FW, Richter M, et al. Multiomic analysis uncovers a continuous spectrum of differentiation and Wnt-MDK-driven immune evasion in hepatoblastoma. J Hepatol. 2025;83(2):367-382. doi:10.1016/j.jhep.2025.01.031

14. Chen J, Bardes EE, Aronow BJ, Jegga AG. ToppGene Suite for gene list enrichment analysis and candidate gene prioritization. Nucleic Acids Research. 2009;37(suppl_2):W305-W311. doi:10.1093/nar/gkp427

15. 10x Genomics. Space Ranger (version 2.1.0). 10x Genomics. <https://www.10xgenomics.com/support/software/space-ranger>

16. 10x Genomics. Loupe Browser (version 8.0.0). 10x Genomics. <https://www.10xgenomics.com/support/software/loupe-browser>

17. Wickham H. ggplot2: Elegant Graphics for Data Analysis. Springer-Verlag New York; 2016. <https://ggplot2.tidyverse.org>

18. Ma RY, Black A, Qian BZ. Macrophage diversity in cancer revisited in the era of single-cell omics. Trends Immunol. 2022;43(7):546-563. doi:10.1016/j.it.2022.04.008

19. Finak G, McDavid A, Yajima M, et al. MAST: a flexible statistical framework for assessing transcriptional changes and characterizing heterogeneity in single-cell RNA sequencing data. Genome Biol. 2015;16(1):278. doi:10.1186/s13059-015-0844-5

20. Jin S, Guerrero-Juarez CF, Zhang L, et al. Inference and analysis of cell-cell communication using CellChat. Nat Commun. 2021;12(1):1088. doi:10.1038/s41467-021-21246-9

21. Jin S, Plikus MV, Nie Q. CellChat for systematic analysis of cell-cell communication from single-cell transcriptomics. Nat Protoc. 2025;20(1):180-219. doi:10.1038/s41596-024-01045-4

22. Thomas C, Soschinski P, Zwaig M, et al. The genetic landscape of choroid plexus tumors in children and adults. Neuro-Oncol. 2021;23(4):650-660. doi:10.1093/neuonc/noaa267

23. Love MI, Huber W, Anders S. Moderated estimation of fold change and dispersion for RNA-seq data with DESeq2. Genome Biol. 2014;15(12):550. doi:10.1186/s13059-014-0550-8

24. Lausen B, Hothorn T, Bretz F, Schumacher M. Assessment of Optimal Selected Prognostic Factors. Biom J. 2004;46(3):364-374. doi:10.1002/bimj.200310030

25. Newman AM, Steen CB, Liu CL, et al. Determining cell type abundance and expression from bulk tissues with digital cytometry. Nat Biotechnol. 2019;37(7):773-782. doi:10.1038/s41587-019-0114-2

26. Therneau TM. survival: A Package for Survival Analysis in R. R package. <https://CRAN.R-project.org/package=survival>

27. Kassambara A, Kosinski M, Biecek P. survminer: Drawing Survival Curves using ‘ggplot2’. R package version 0.5.1. <https://CRAN.R-project.org/package=survminer>
